# A retrospective analysis of ketamine administration by critical care paramedics in a pre-hospital care setting

**DOI:** 10.29045/14784726.2018.03.2.4.25

**Published:** 2018-03-01

**Authors:** Alan Cowley, Julia Williams, Pete Westhead, Nick Gray, Adam Watts, Fionna Moore

**Affiliations:** South East Coast Ambulance Service NHS Foundation Trust; South East Coast Ambulance Service NHS Foundation Trust; Brighton and Sussex University Hospitals NHS Trust; South East Coast Ambulance Service NHS Foundation Trust; South East Coast Ambulance Service NHS Foundation Trust; South East Coast Ambulance Service NHS Foundation Trust

**Keywords:** analgesia, emergency medical technicians, ketamine

## Abstract

**Objective:**

This project aims to describe pre-hospital use of ketamine in trauma by South East Coast Ambulance Service critical care paramedics and evaluate the occurrence of any side effects or adverse events.

**Methods:**

A retrospective analysis of patients receiving pre-hospital ketamine for trauma between 16 March 2013 and 30 April 2017. Administrations were identified from Advanced Life Saving Interventions and Procedures reports submitted by the clinician and, later, from an electronic database. Each was scrutinised for patient demographics, doses and reports of side effects or adverse events.

**Results:**

A total of 510 unique administrations were identified. Following the exclusion of 61 records, 449 (88.0%) administrations remained. The most common indication for administration of ketamine was lower limb injury, with 228 (50.8%) administrations. Ketamine was only administered intravenously, and the median dose of ketamine for all administrations was 30 mg (interquartile range 20–40 mg). The gender split was dominated by males who accounted for 302 (67.3%) administrations compared to 147 (32.7%) females. The median age of patients was 44 years (interquartile range 28–58 years), with women on average being older than men. Telephone calls to a consultant were made for 243/449 (54.1%) of the administrations, reflecting a need for sanctioning of the drug, advice on dosages or indications, for example.

**Conclusions:**

Critical care paramedics within a well governed system are able to safely administer ketamine within an approved dosing regimen under a Patient Group Direction. Median doses are in keeping with nationally approved guidelines. Reported side effects were within the described frequencies in the British National Formulary. Prospective studies are now needed in order to confirm the safety and efficacy of ketamine administration among the advanced paramedic population.



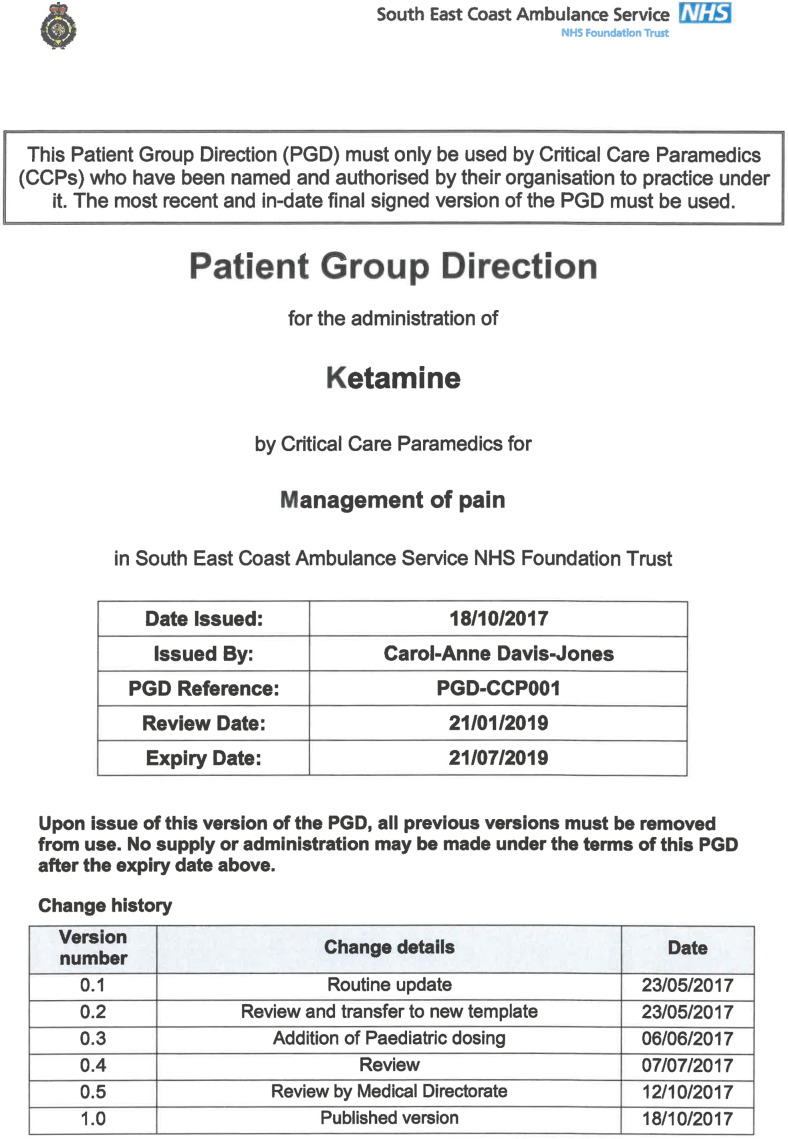



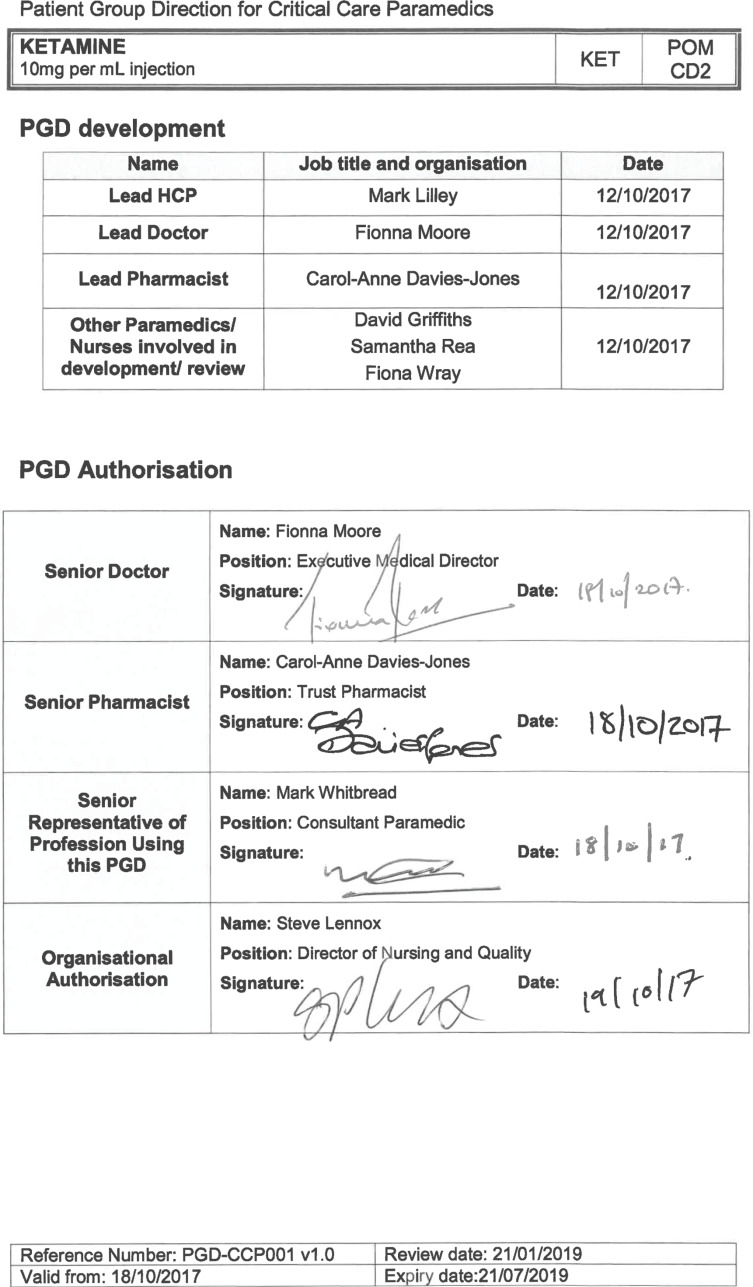



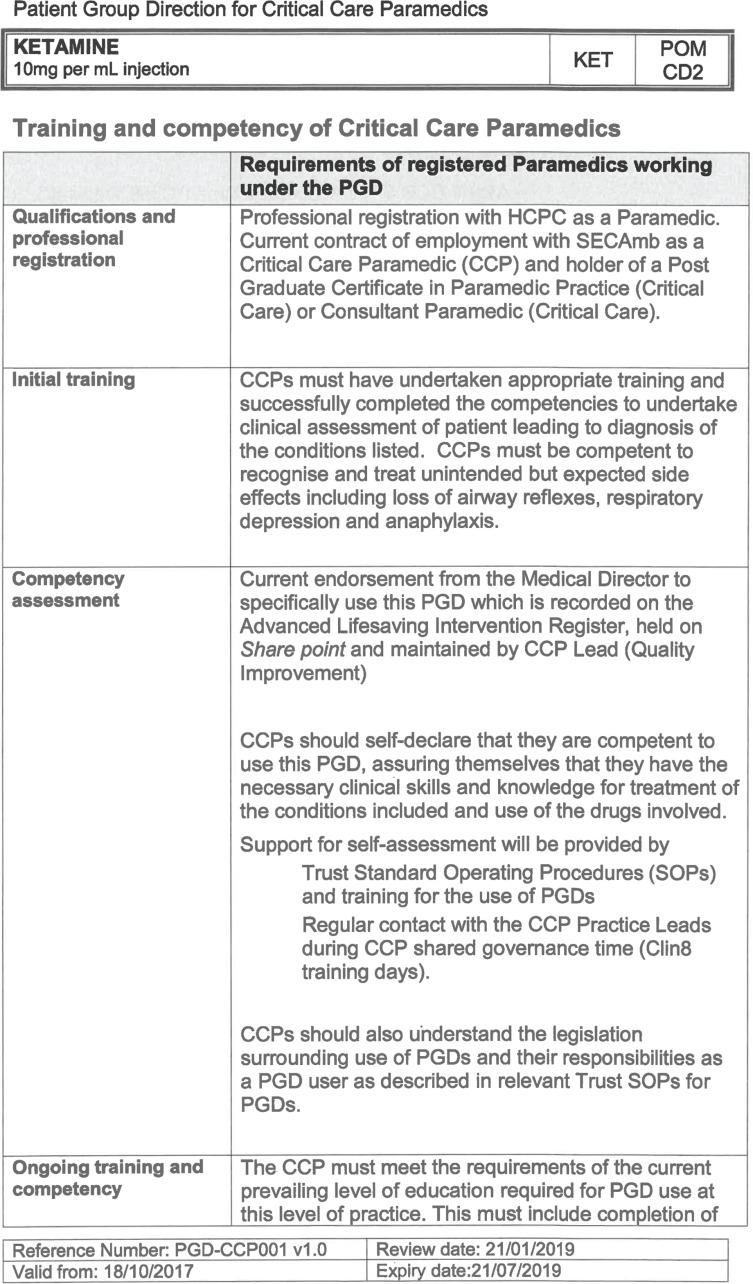



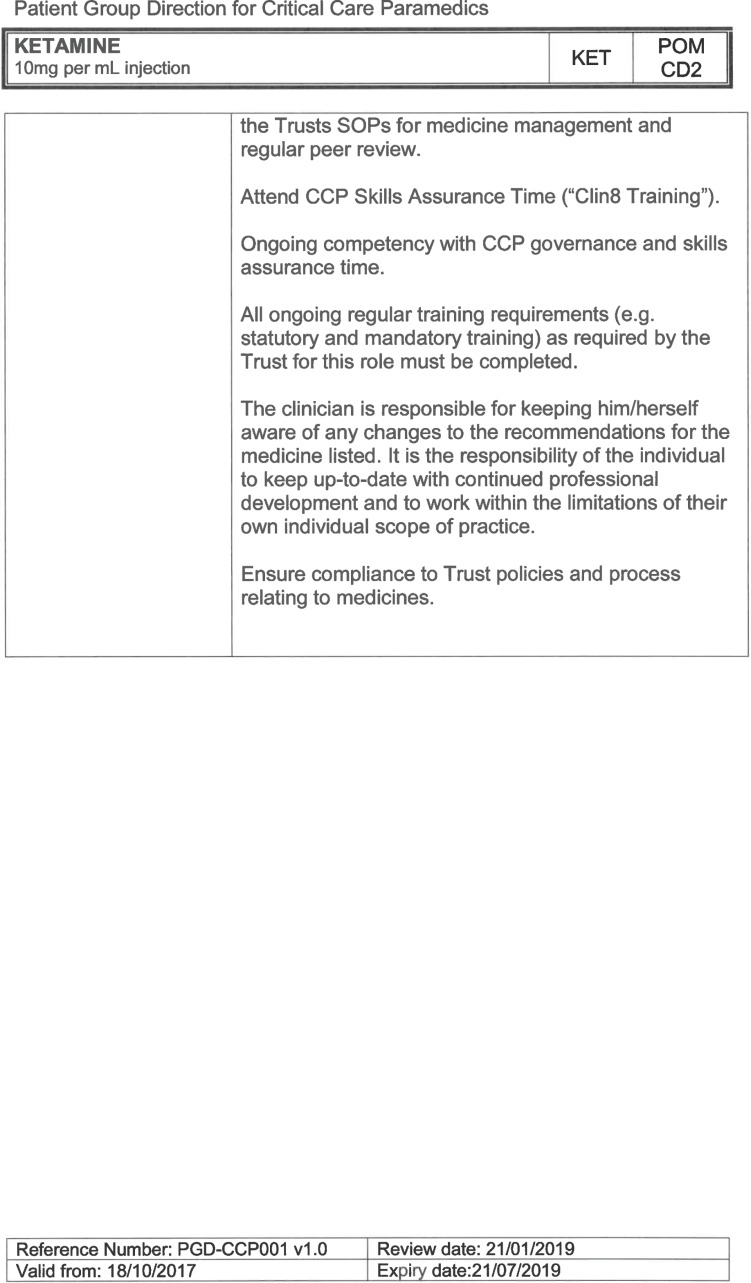



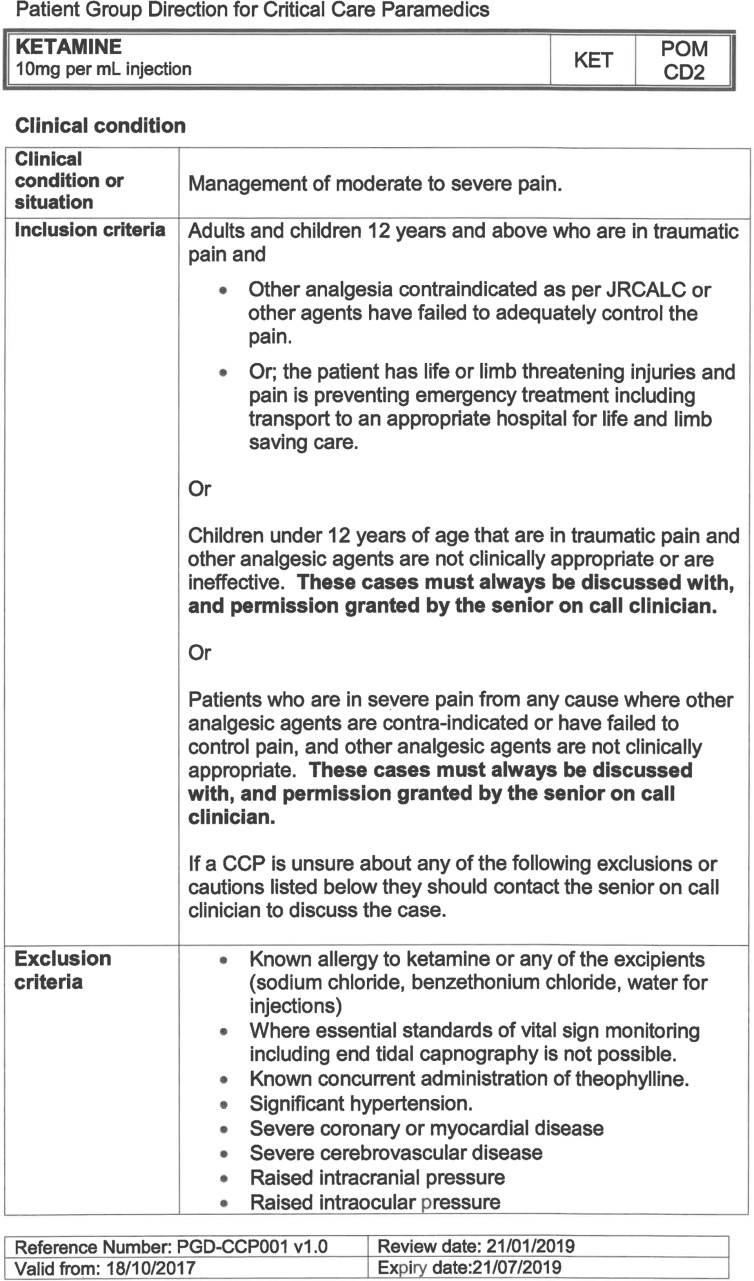



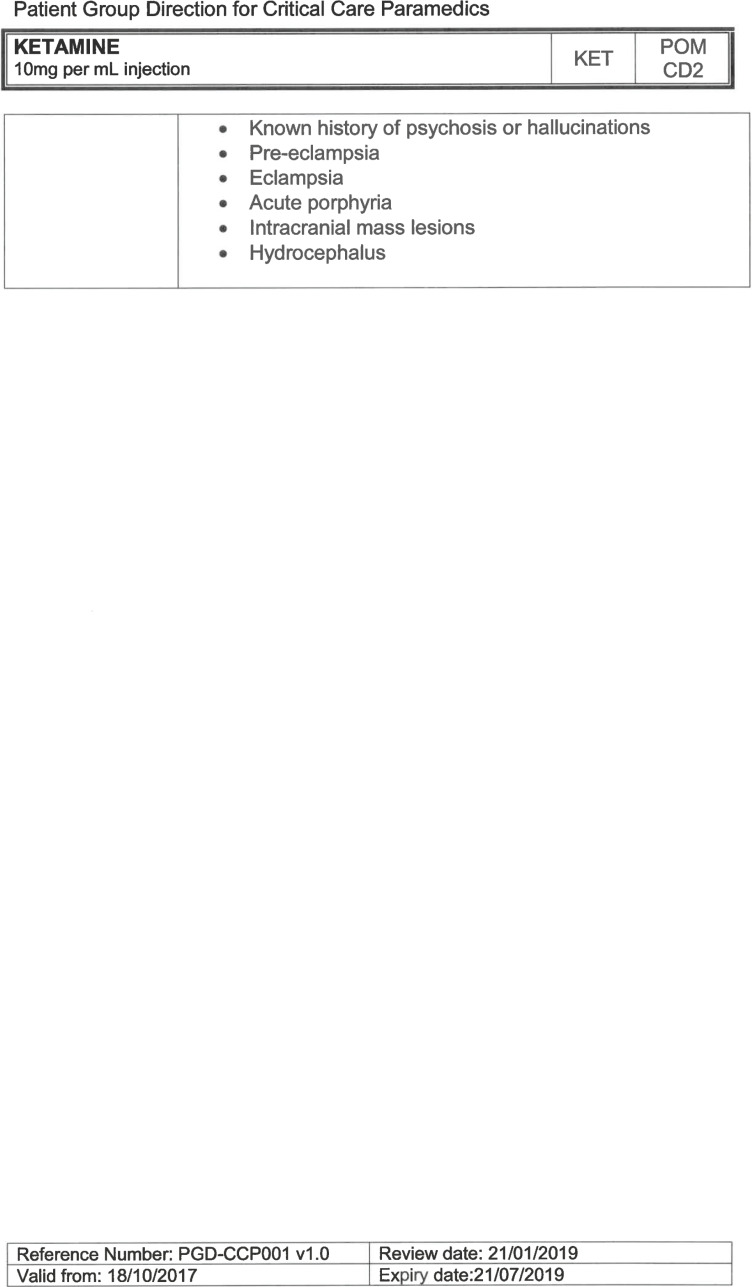



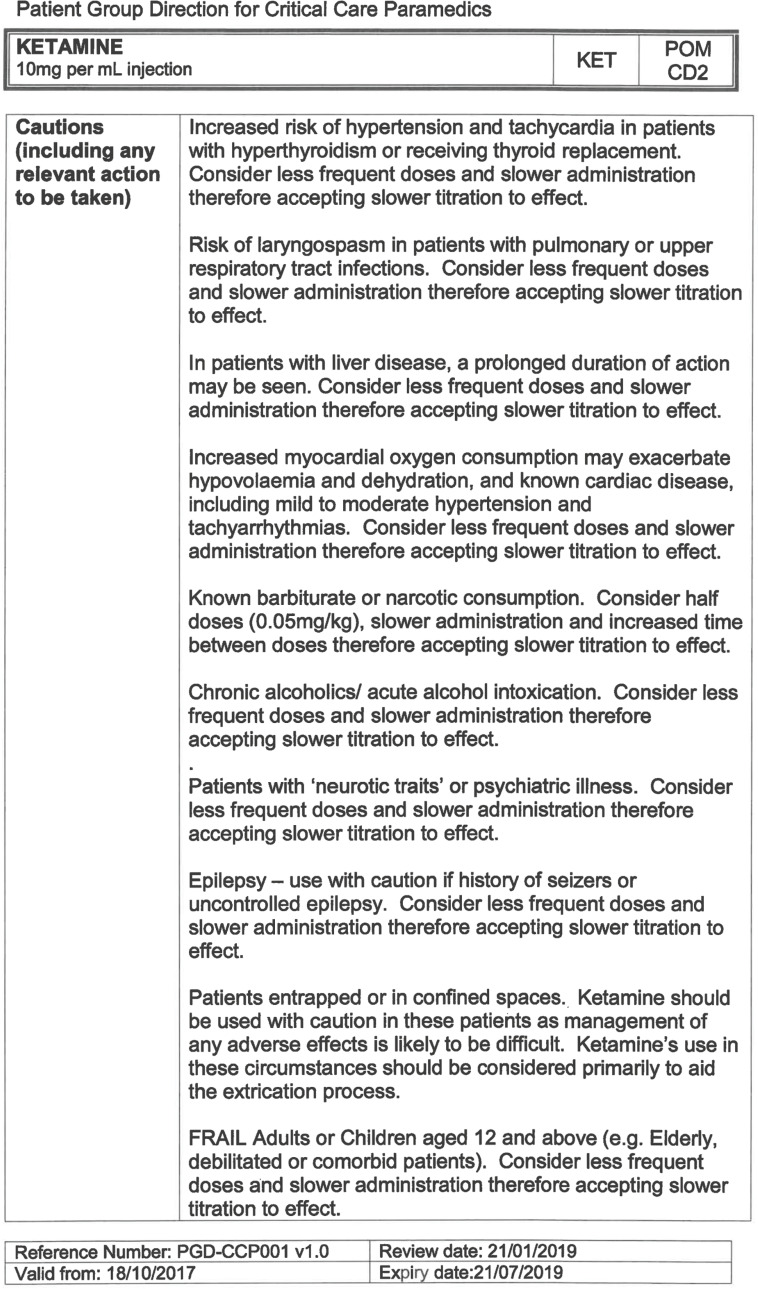



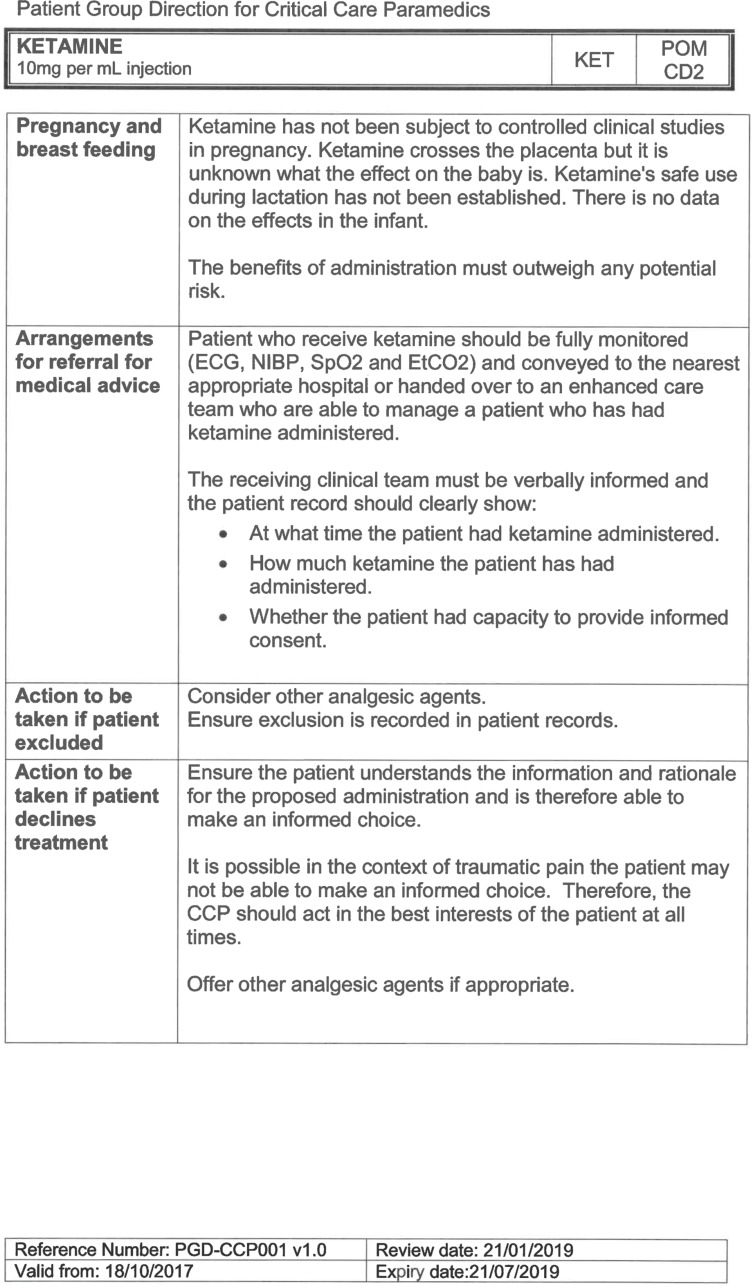



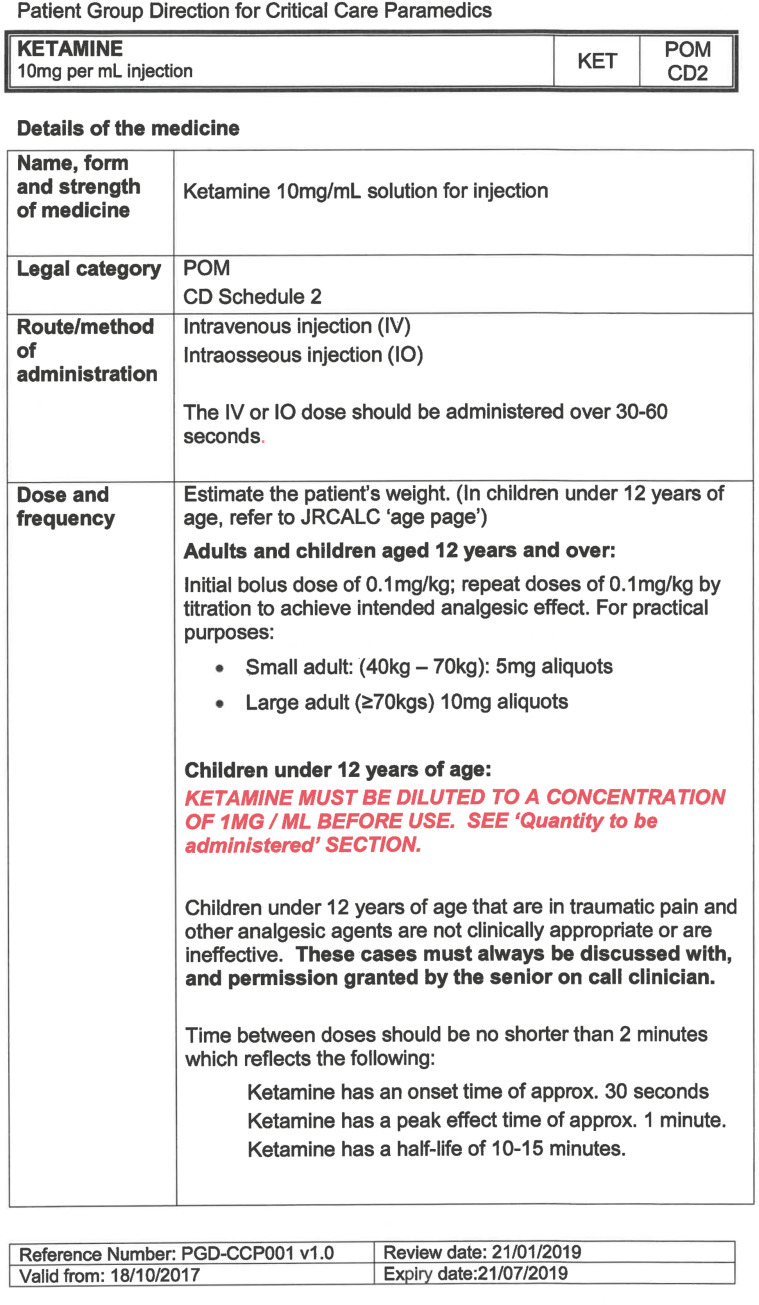



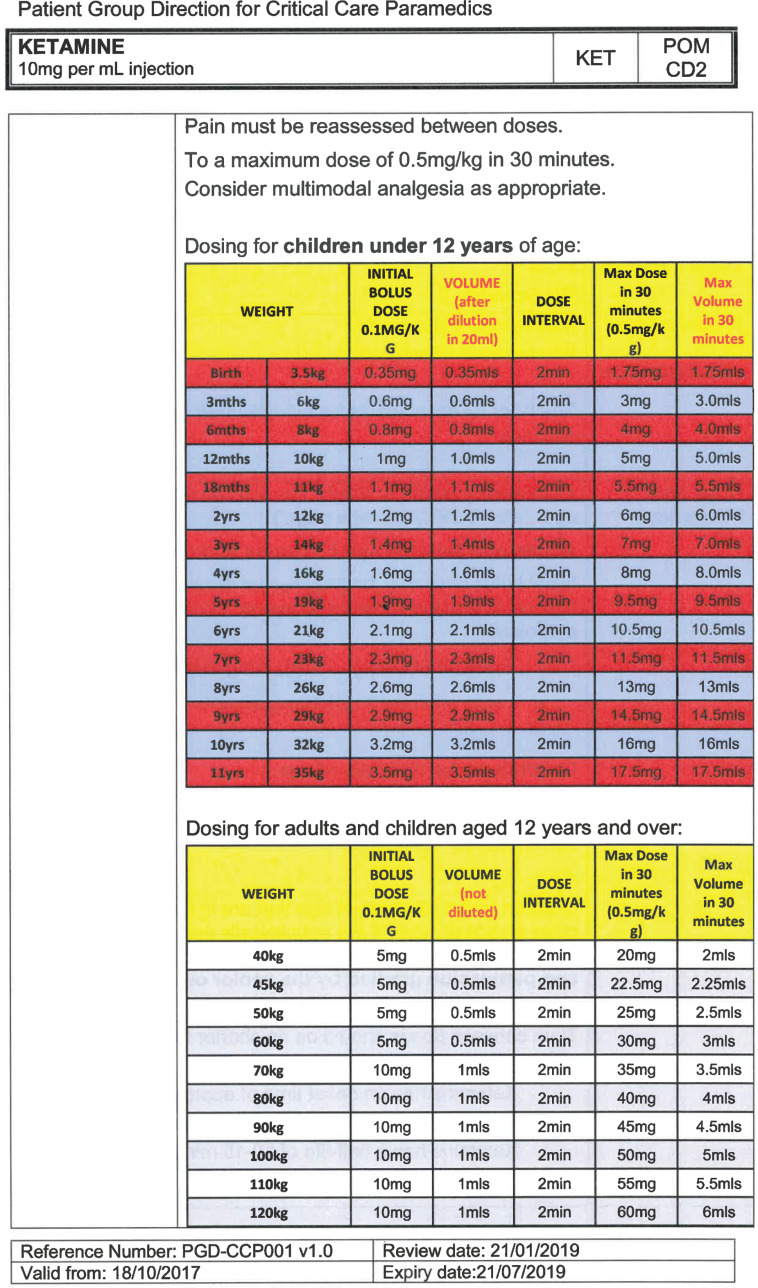



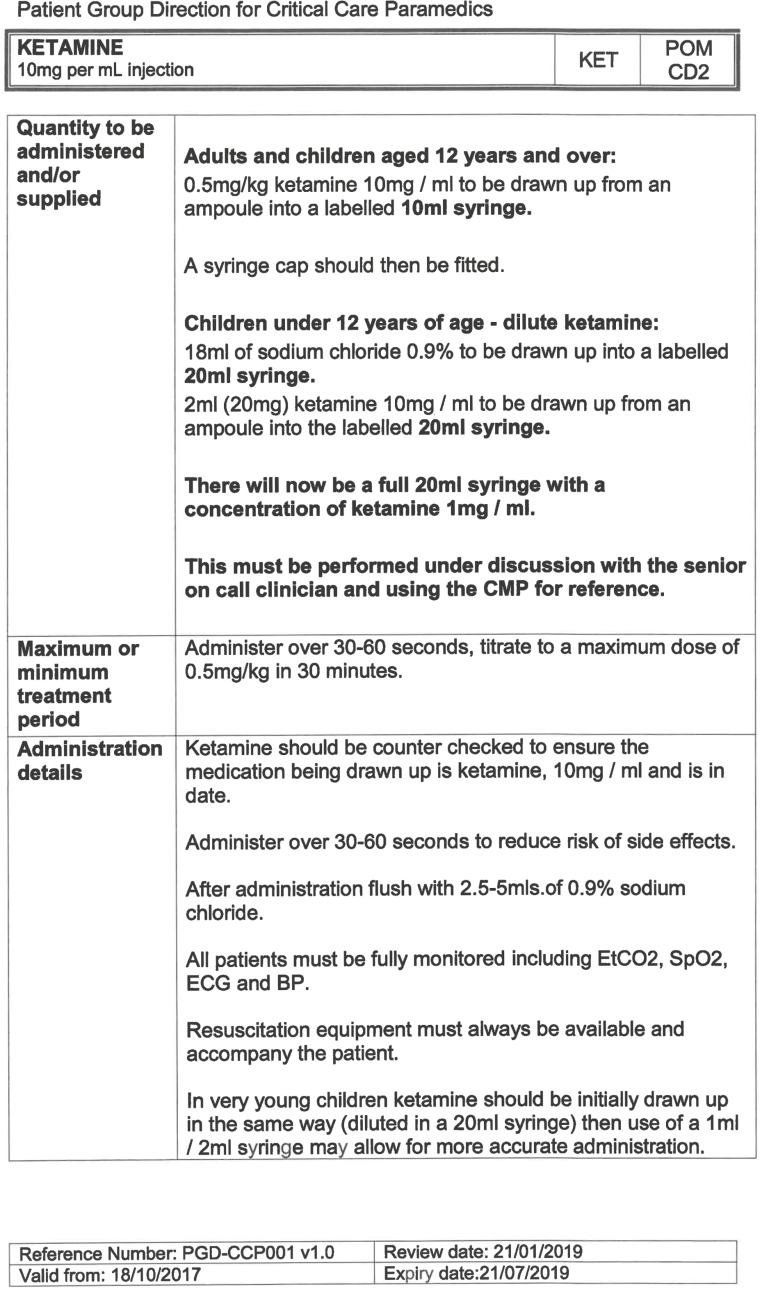



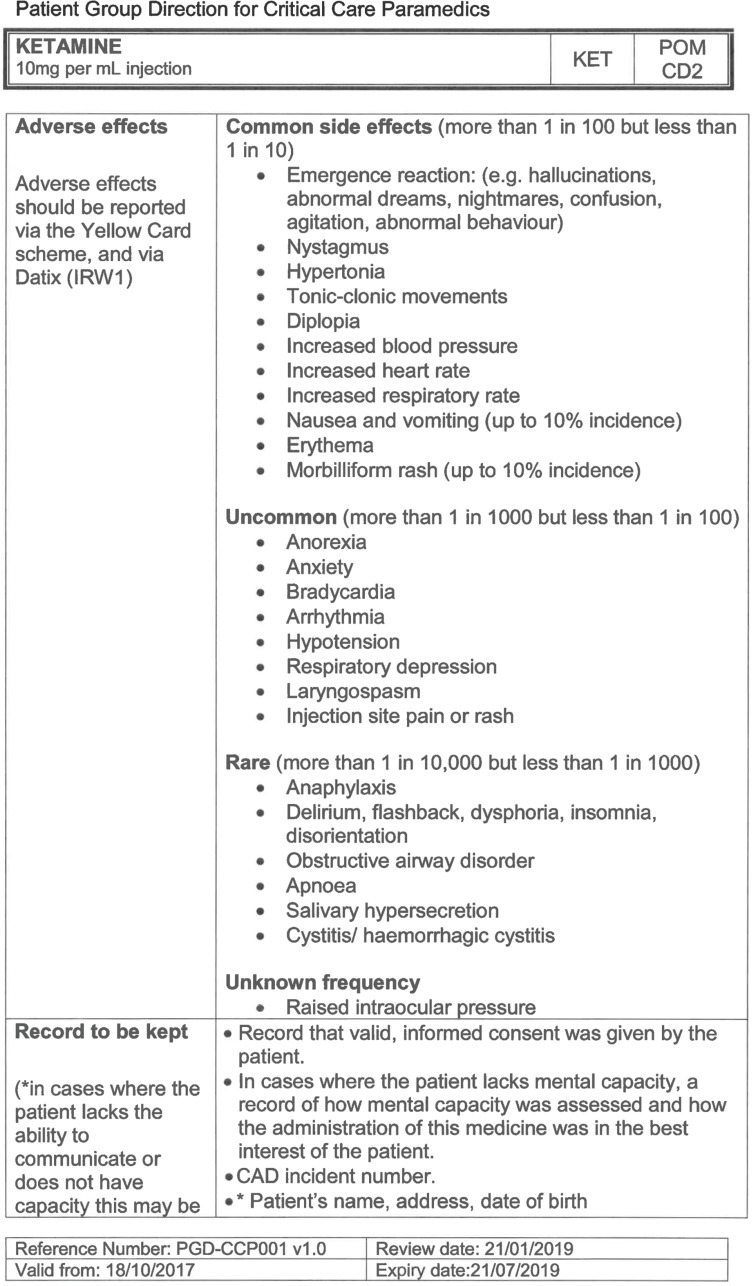



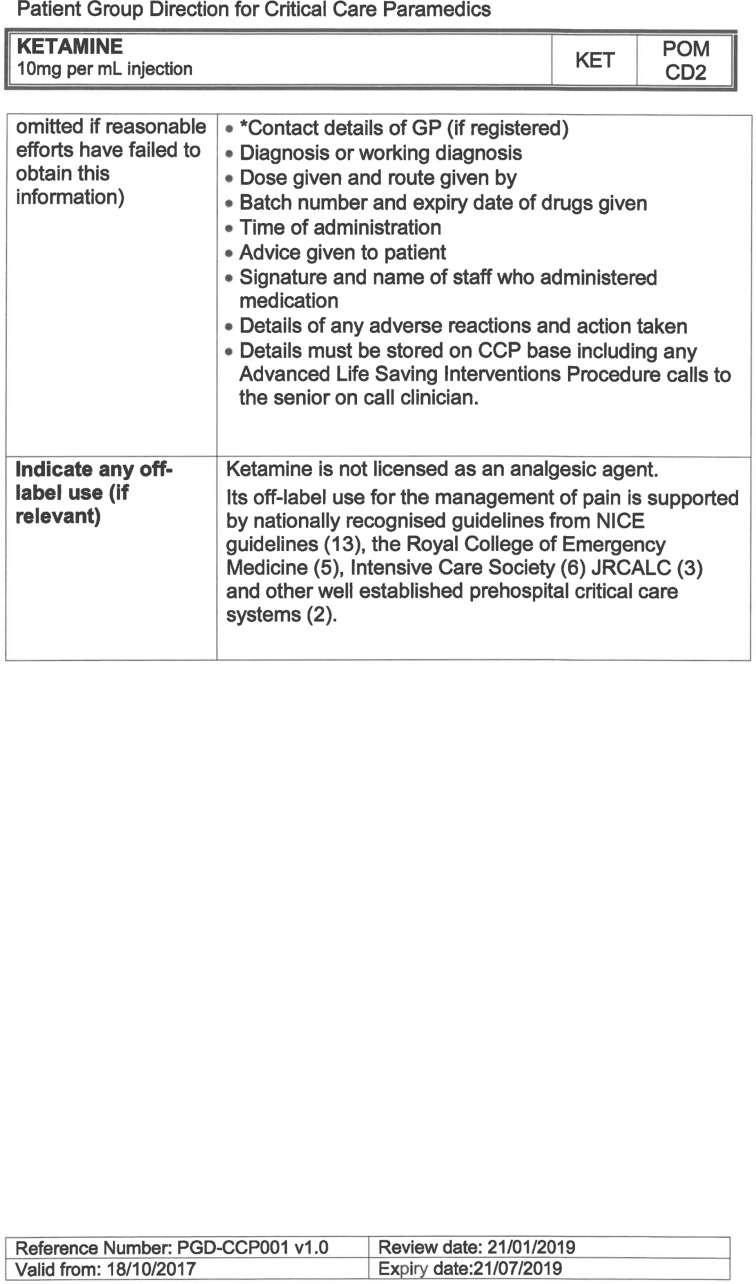



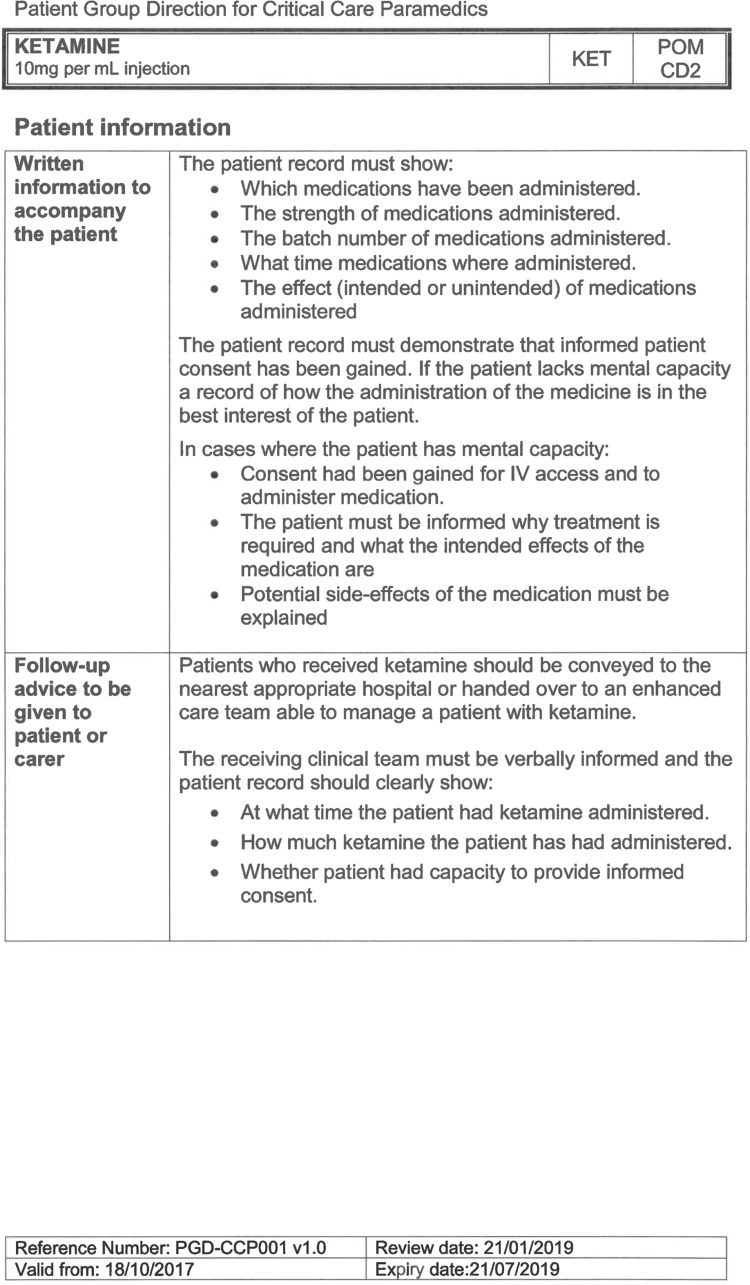



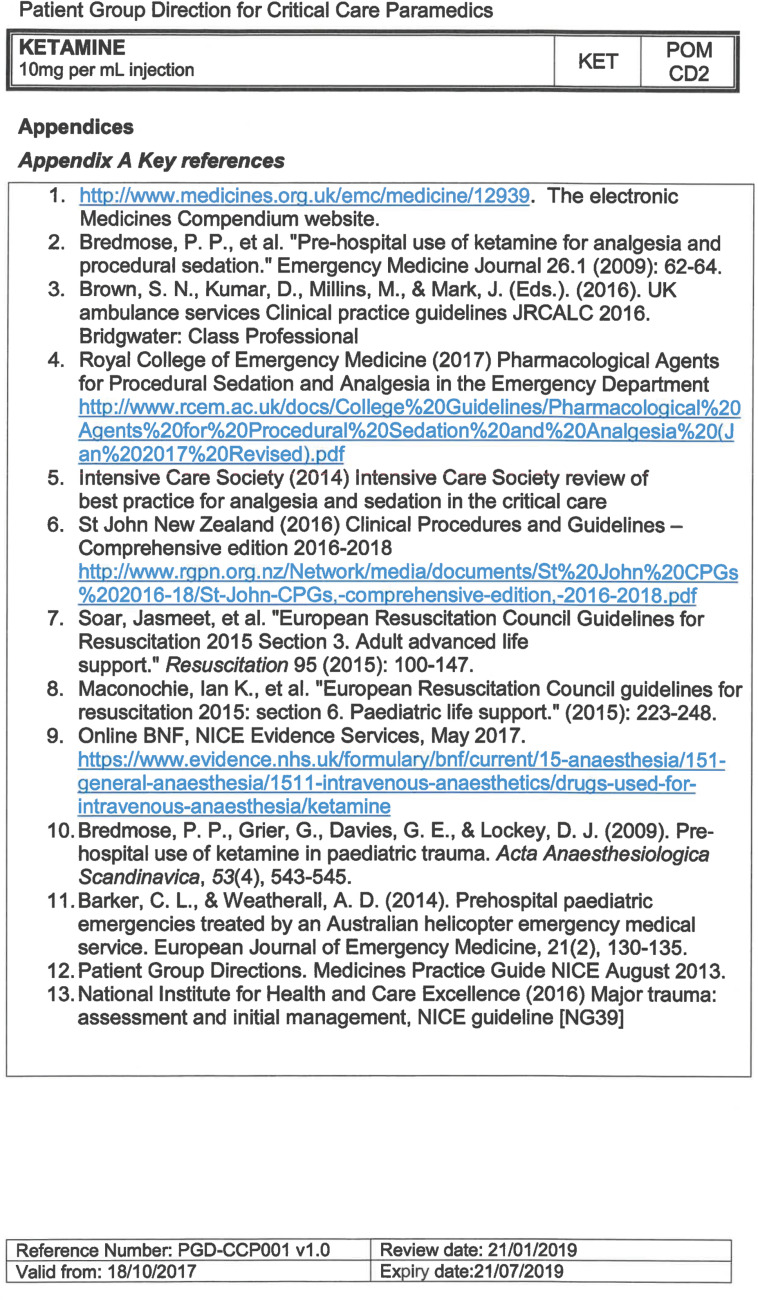



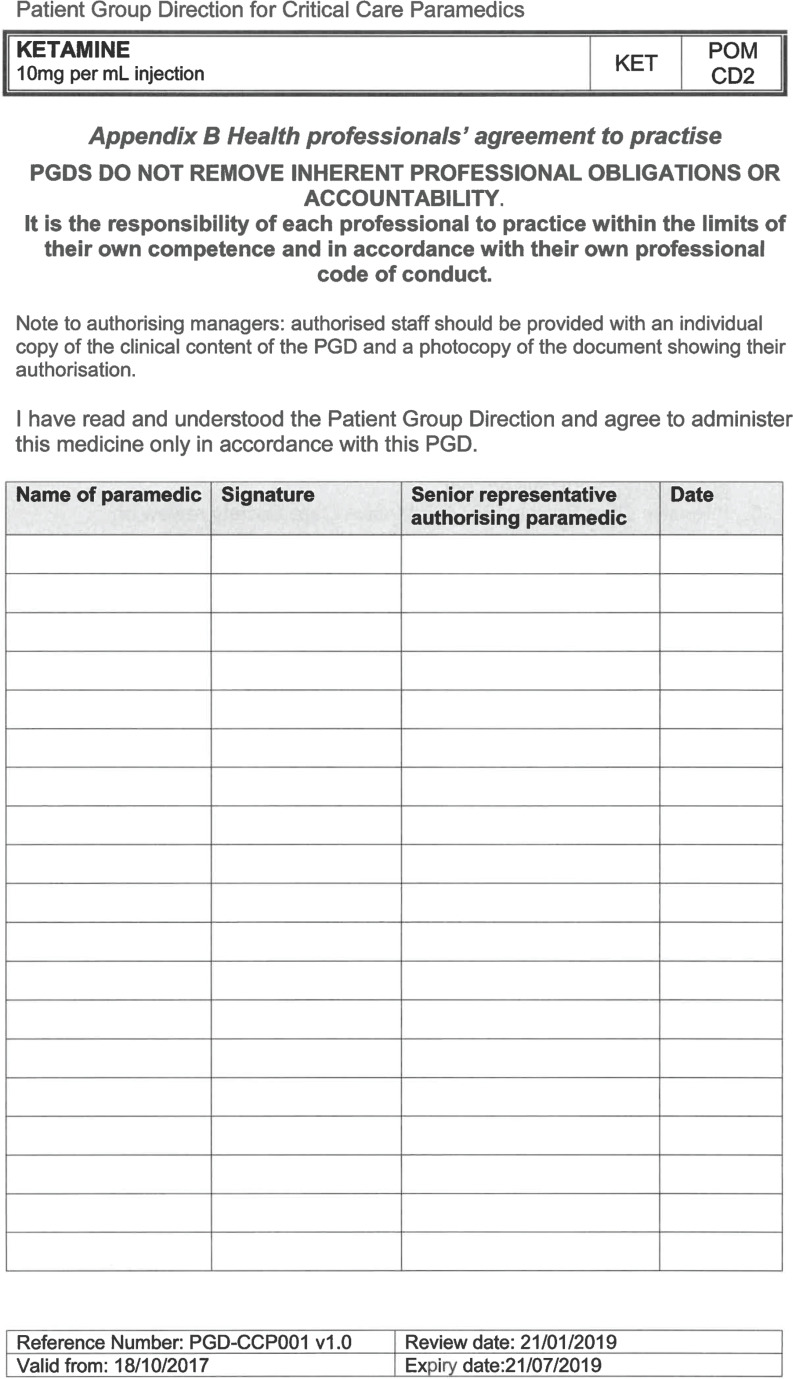

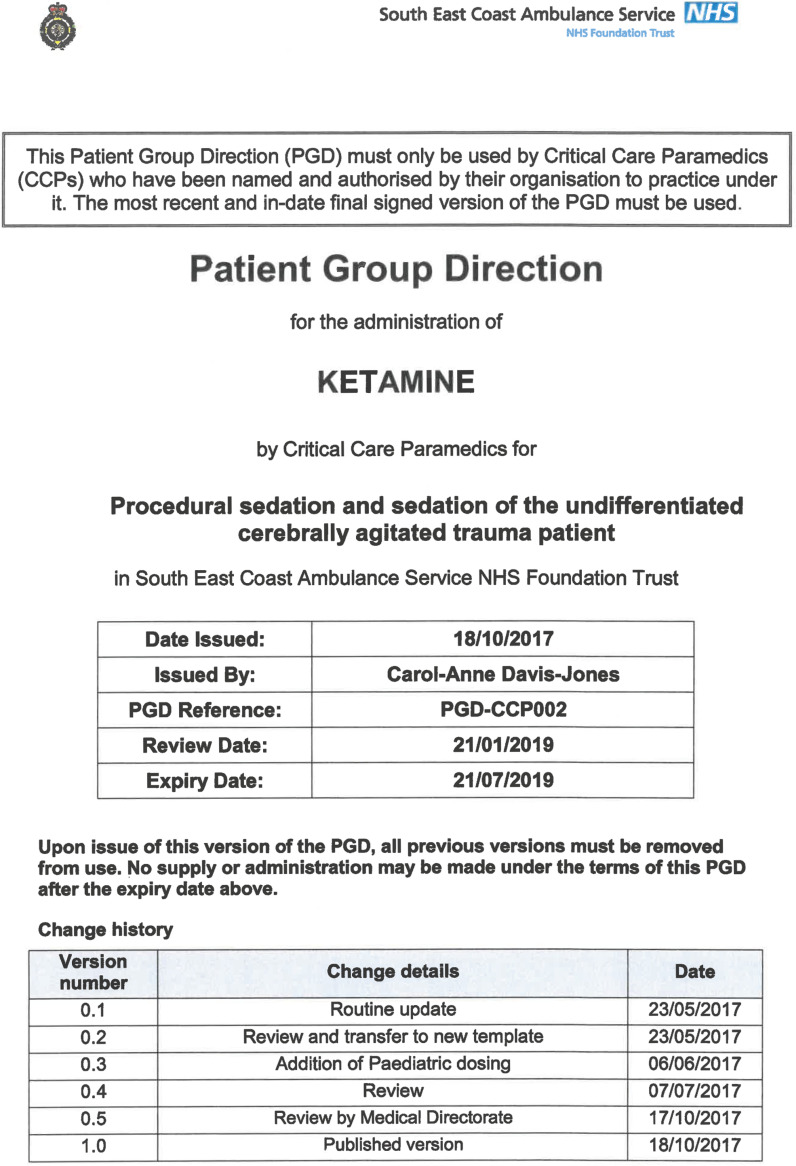



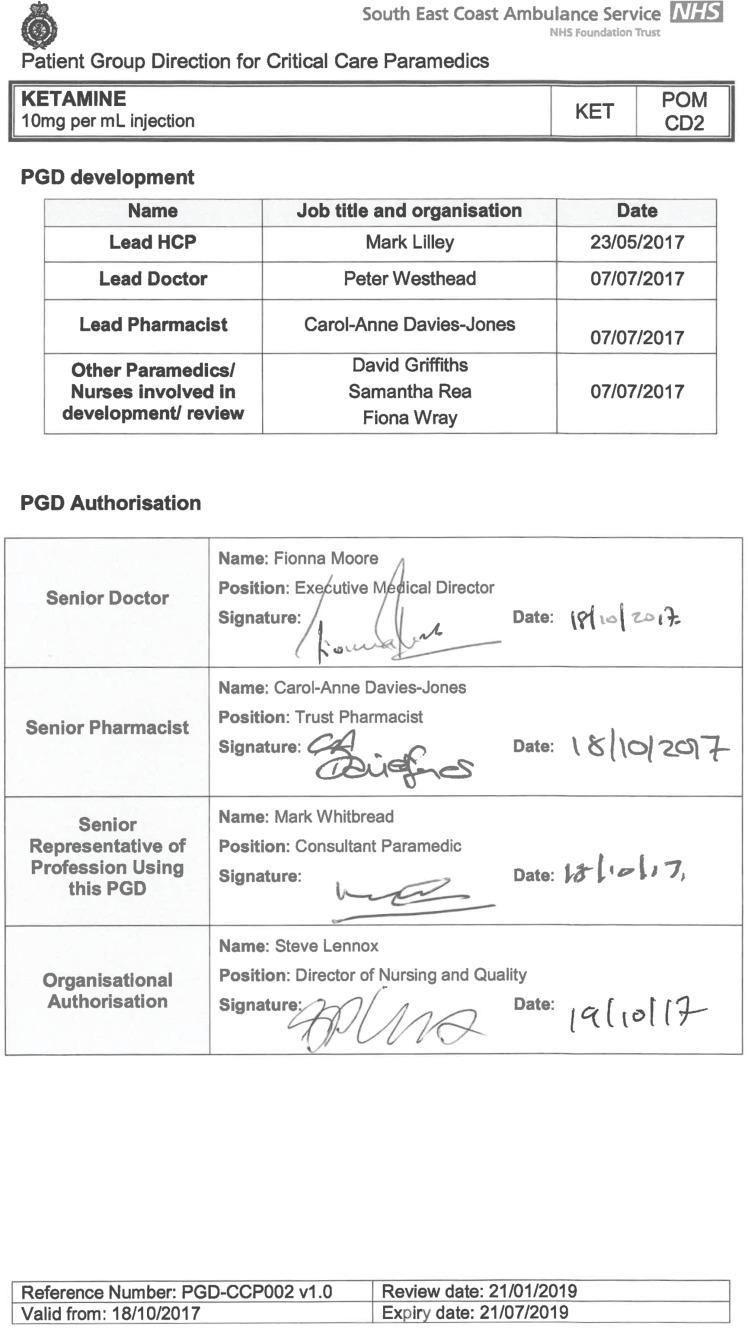



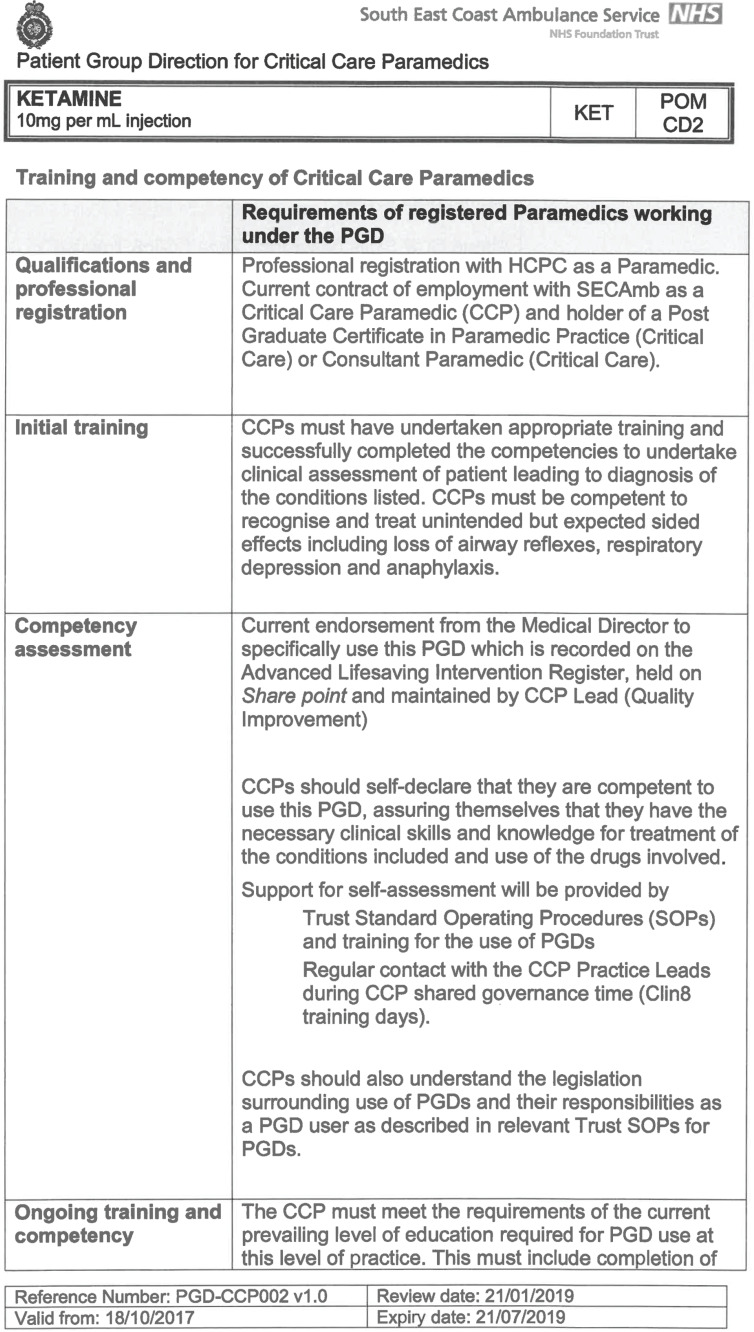



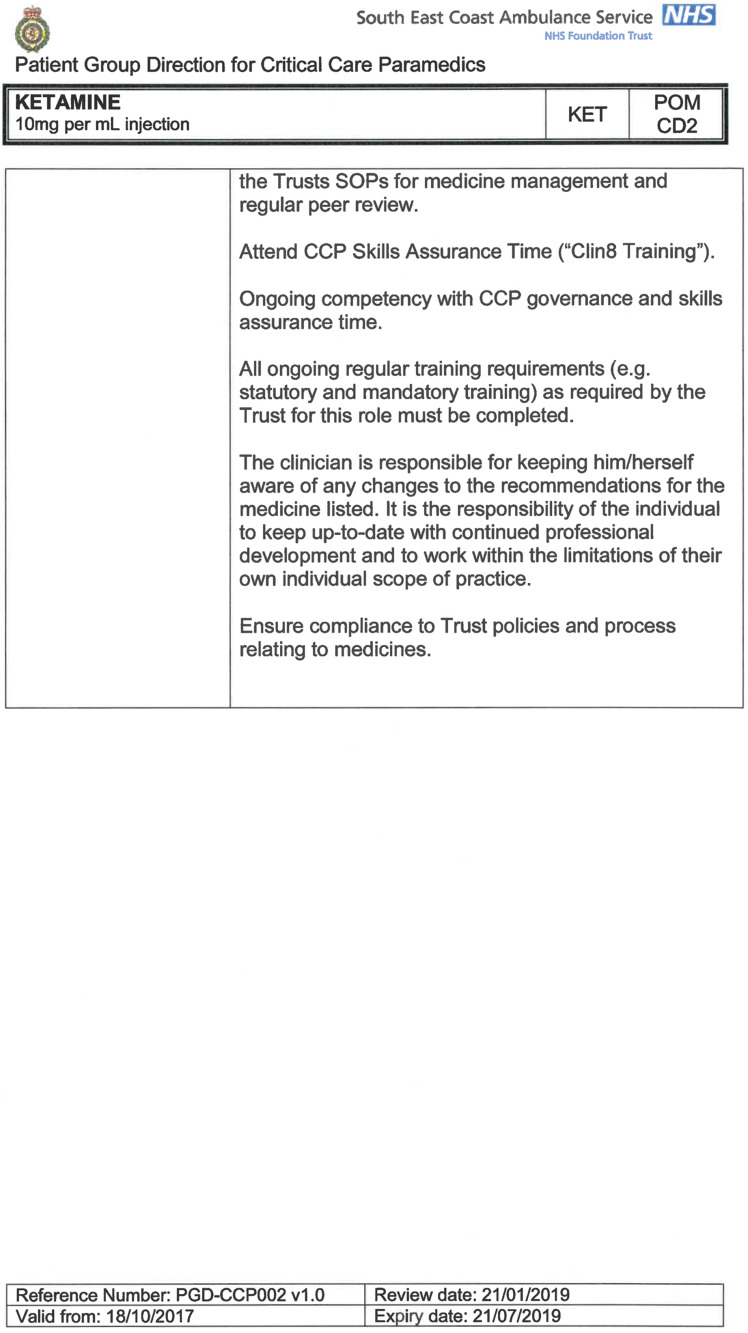



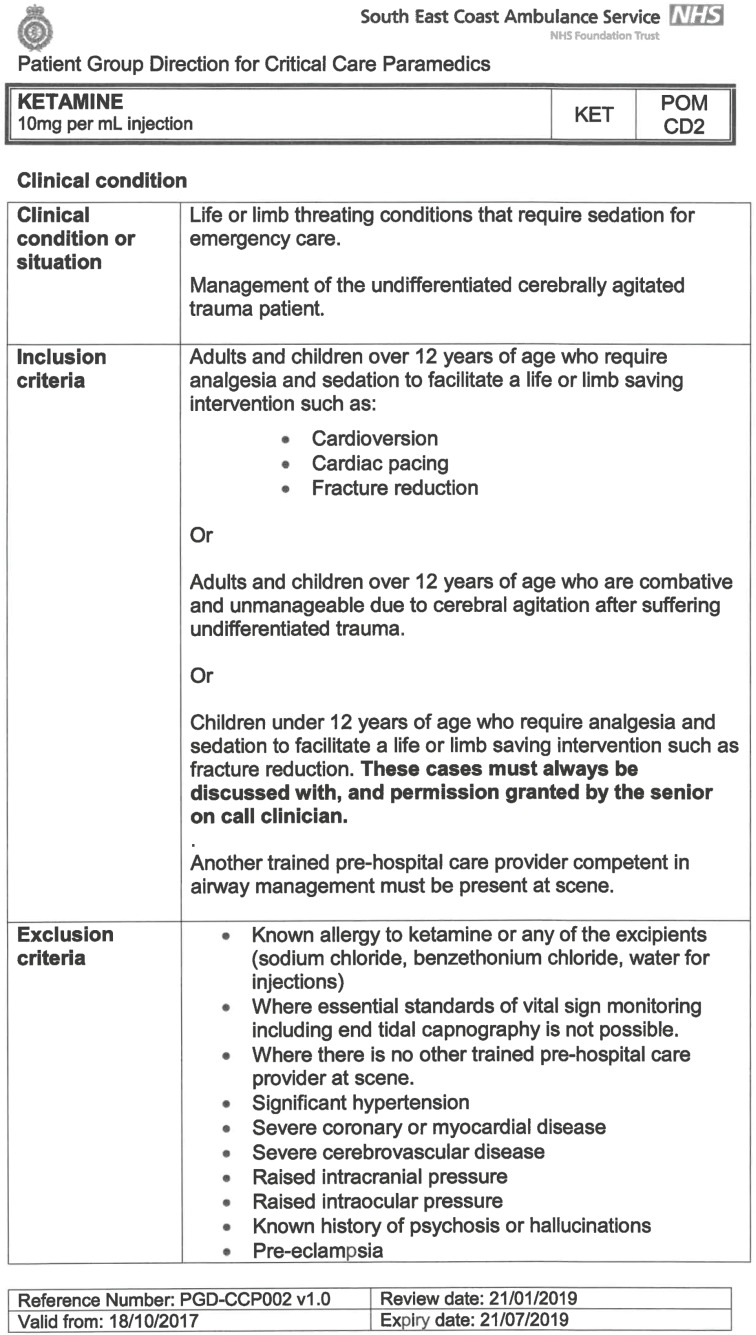



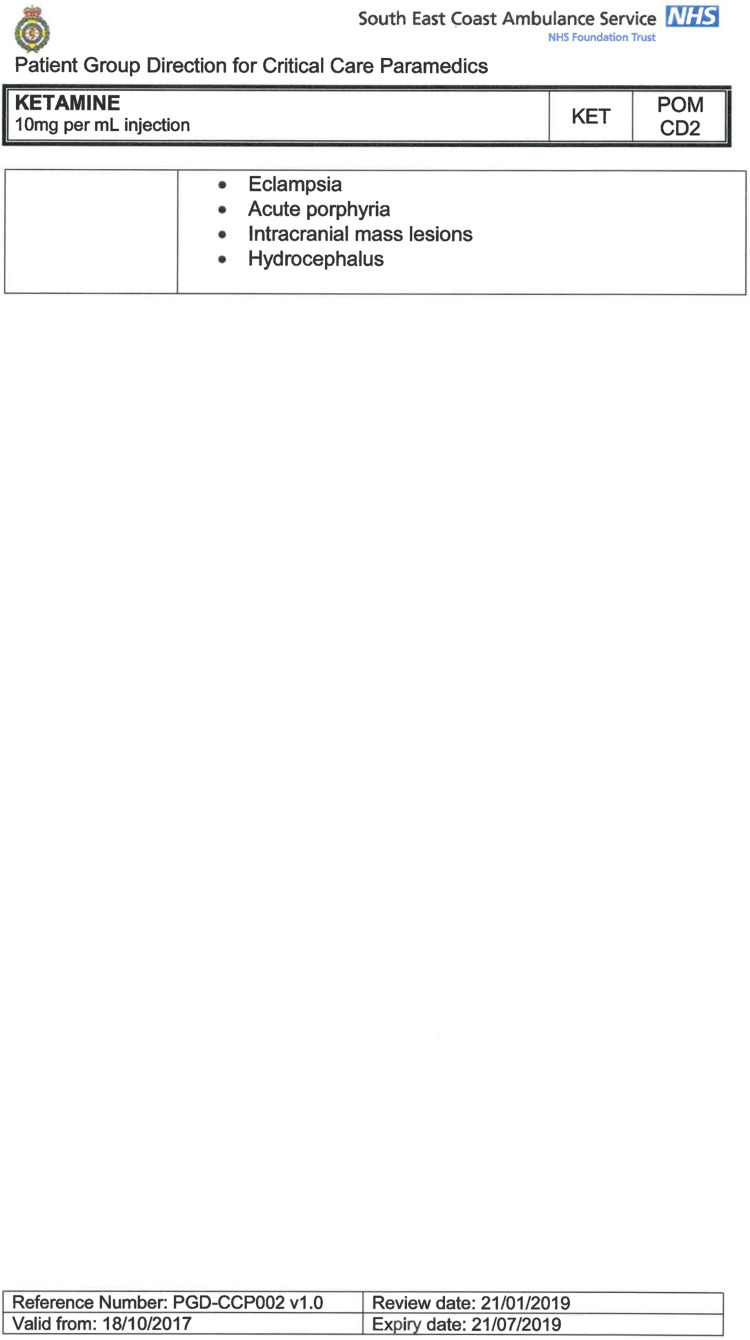



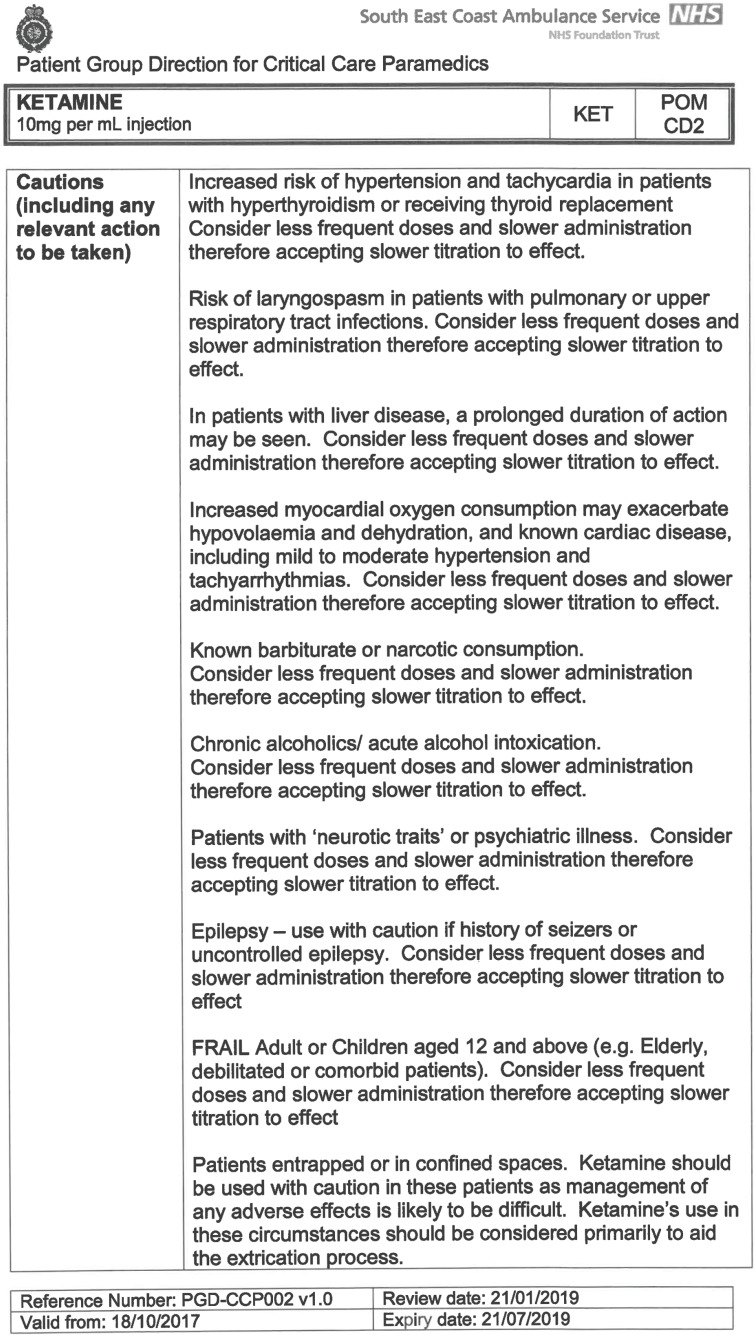



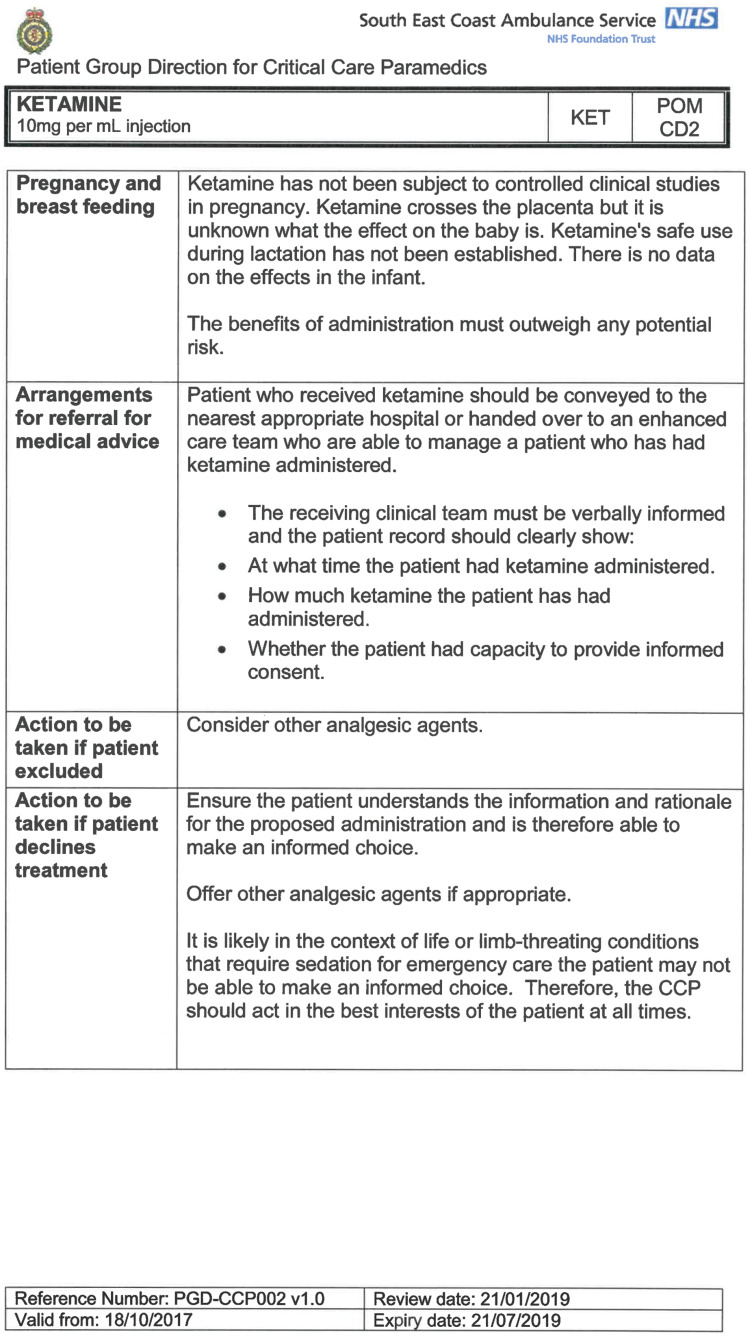



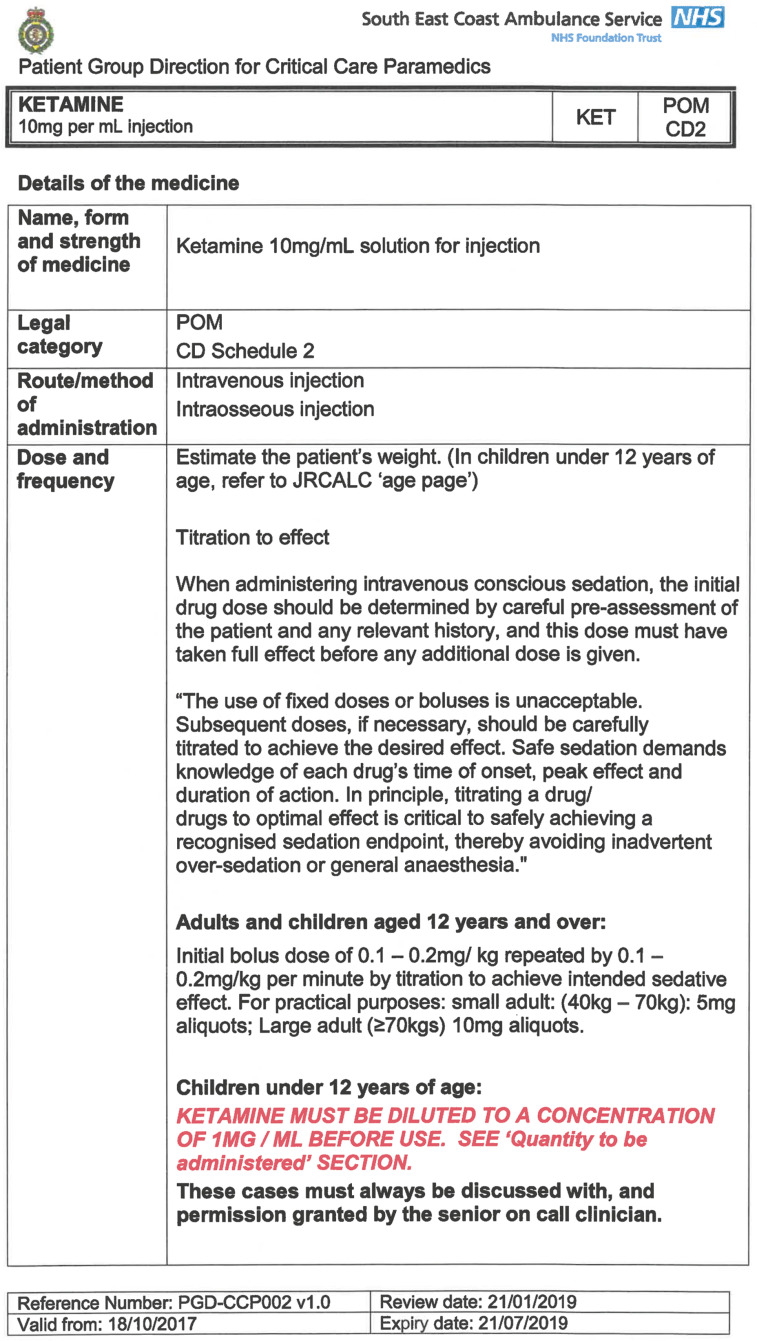



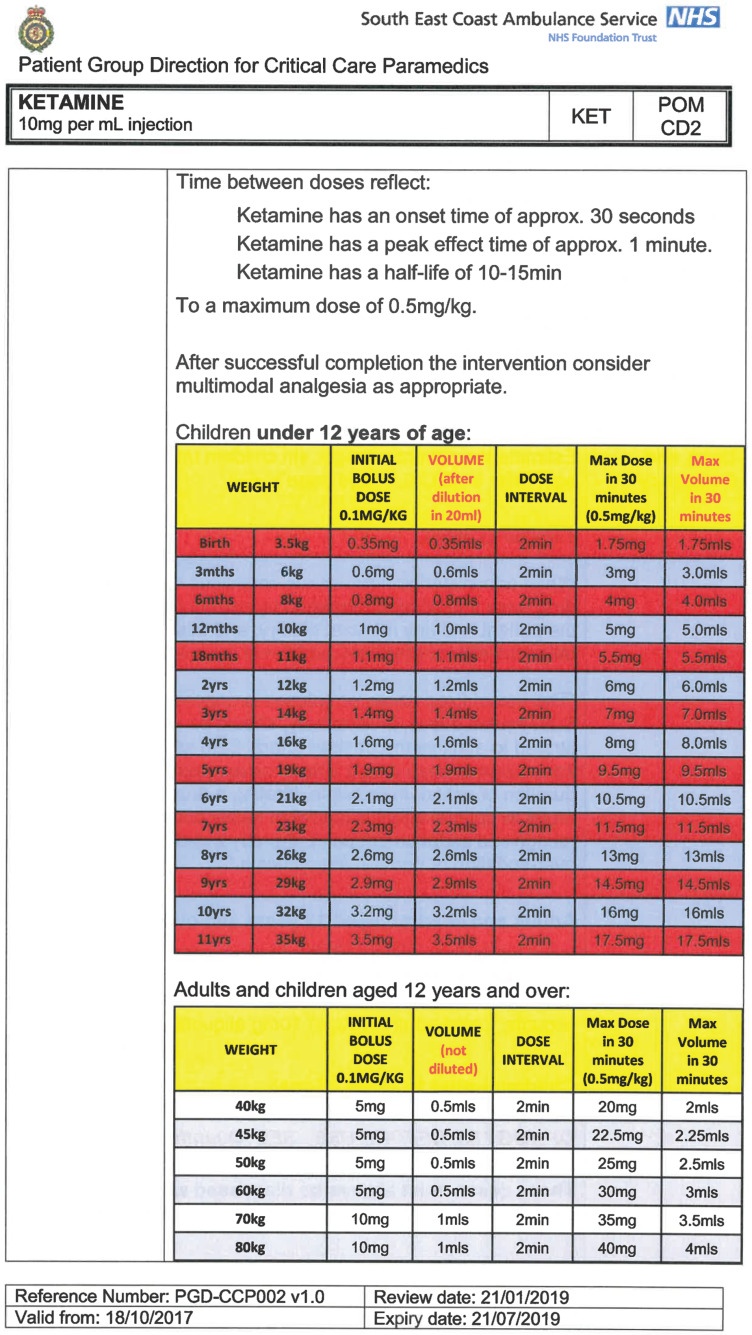



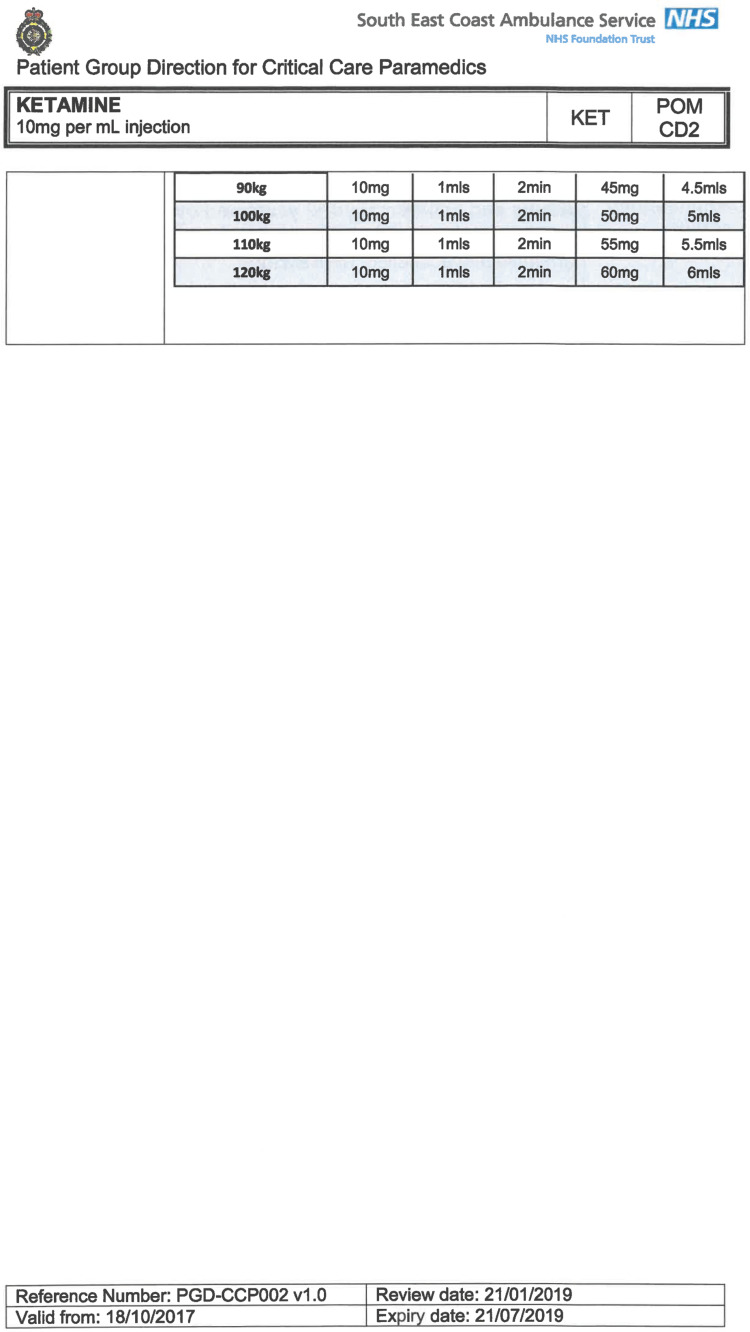



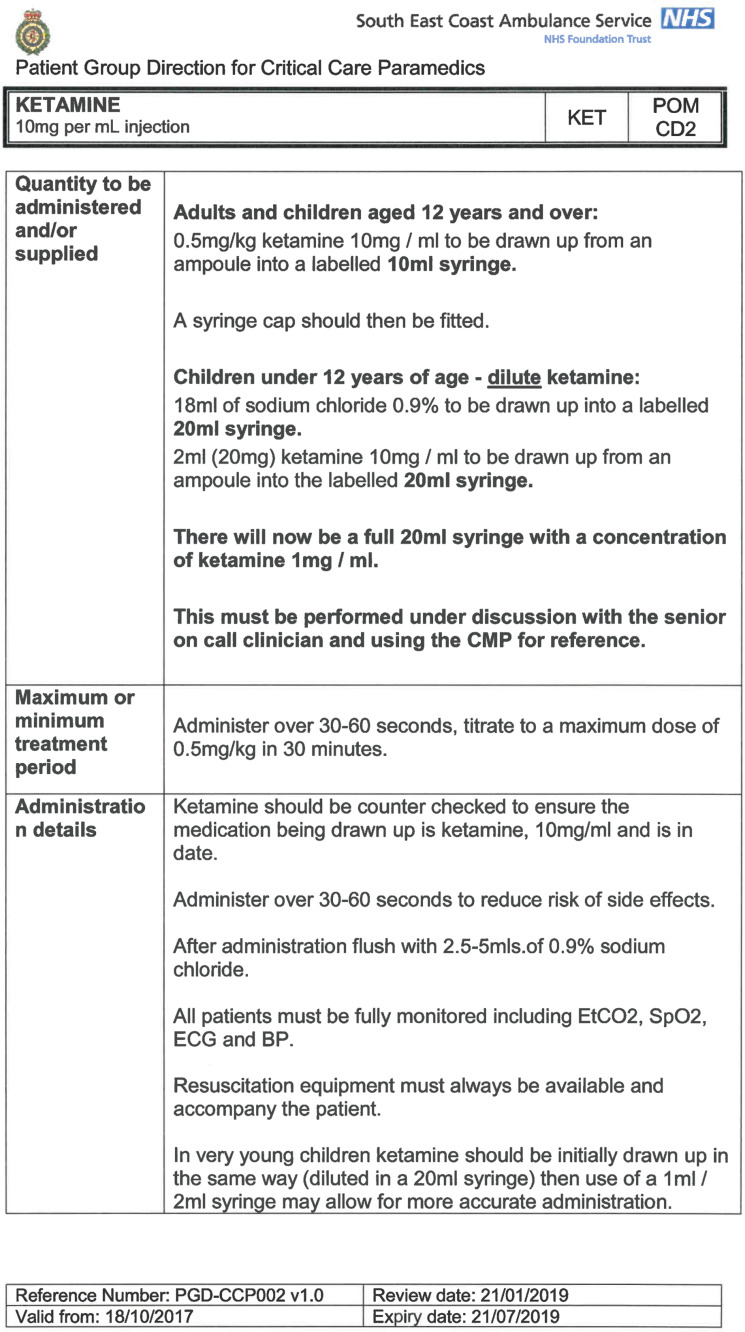



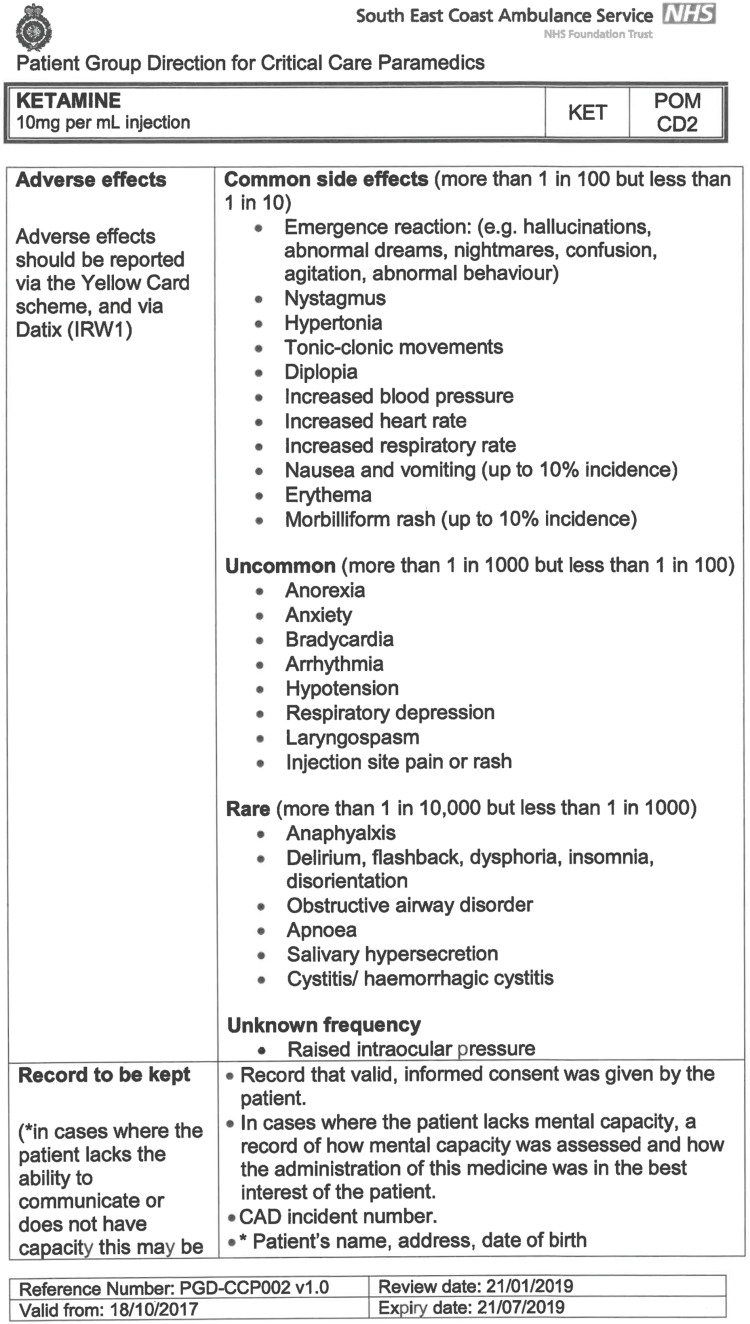



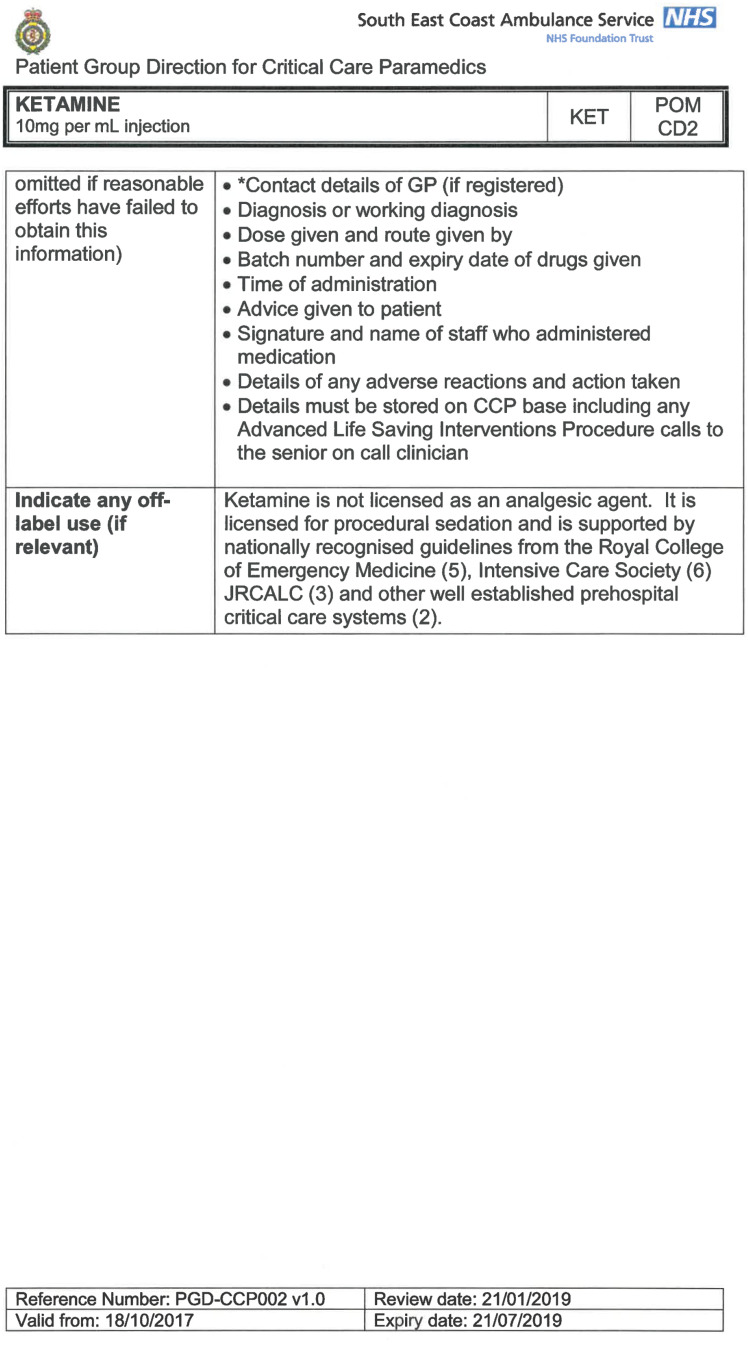



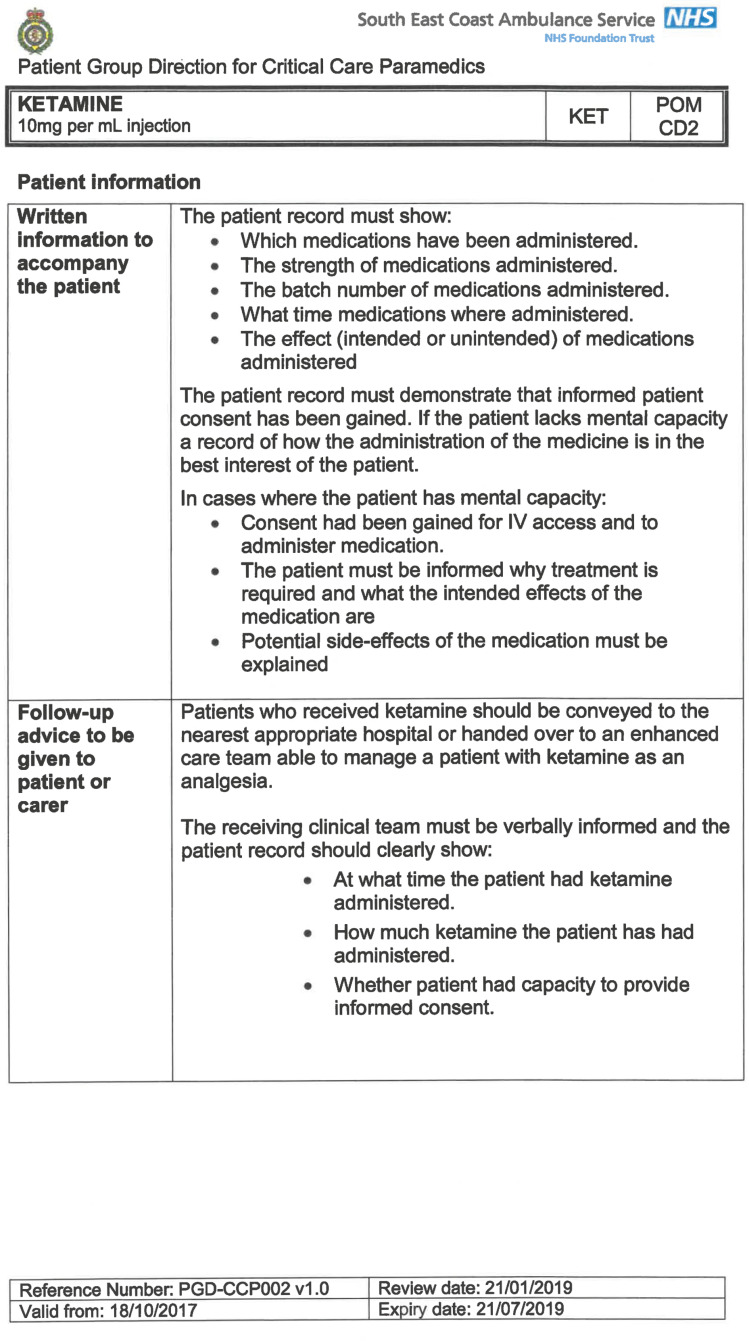



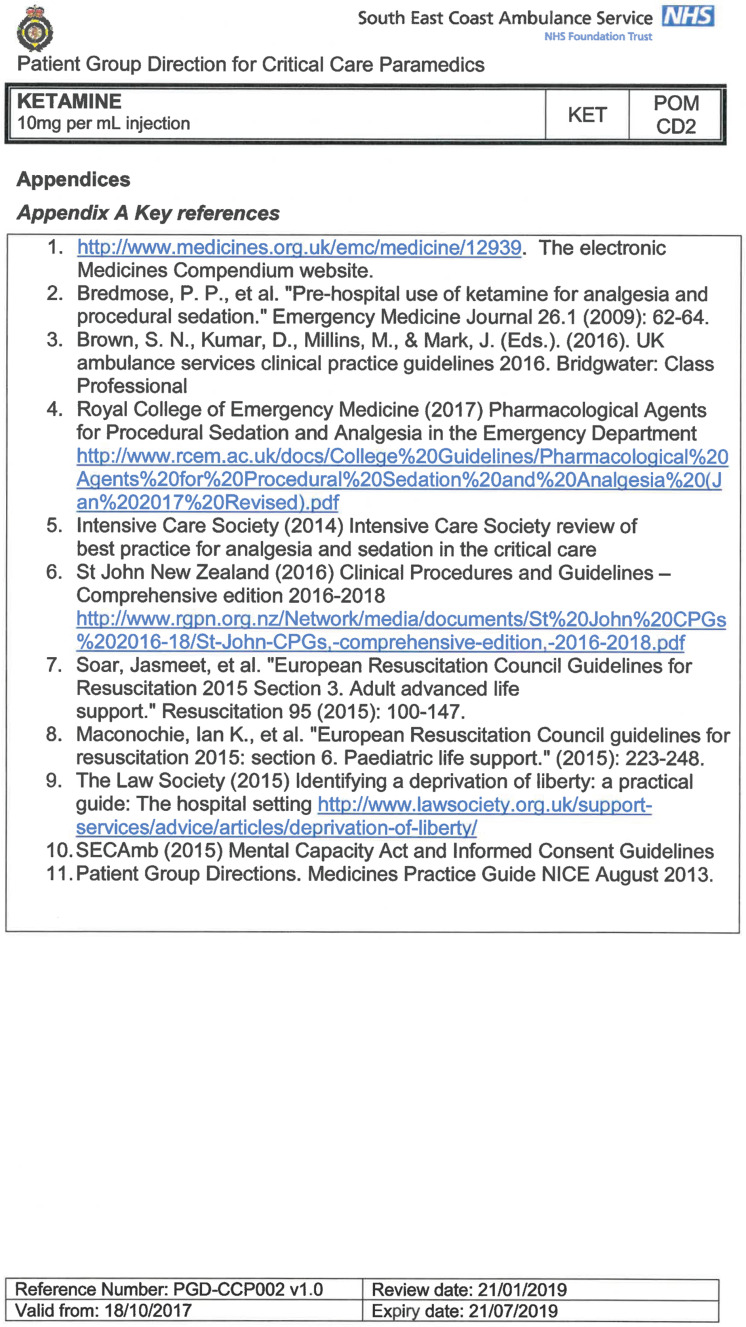



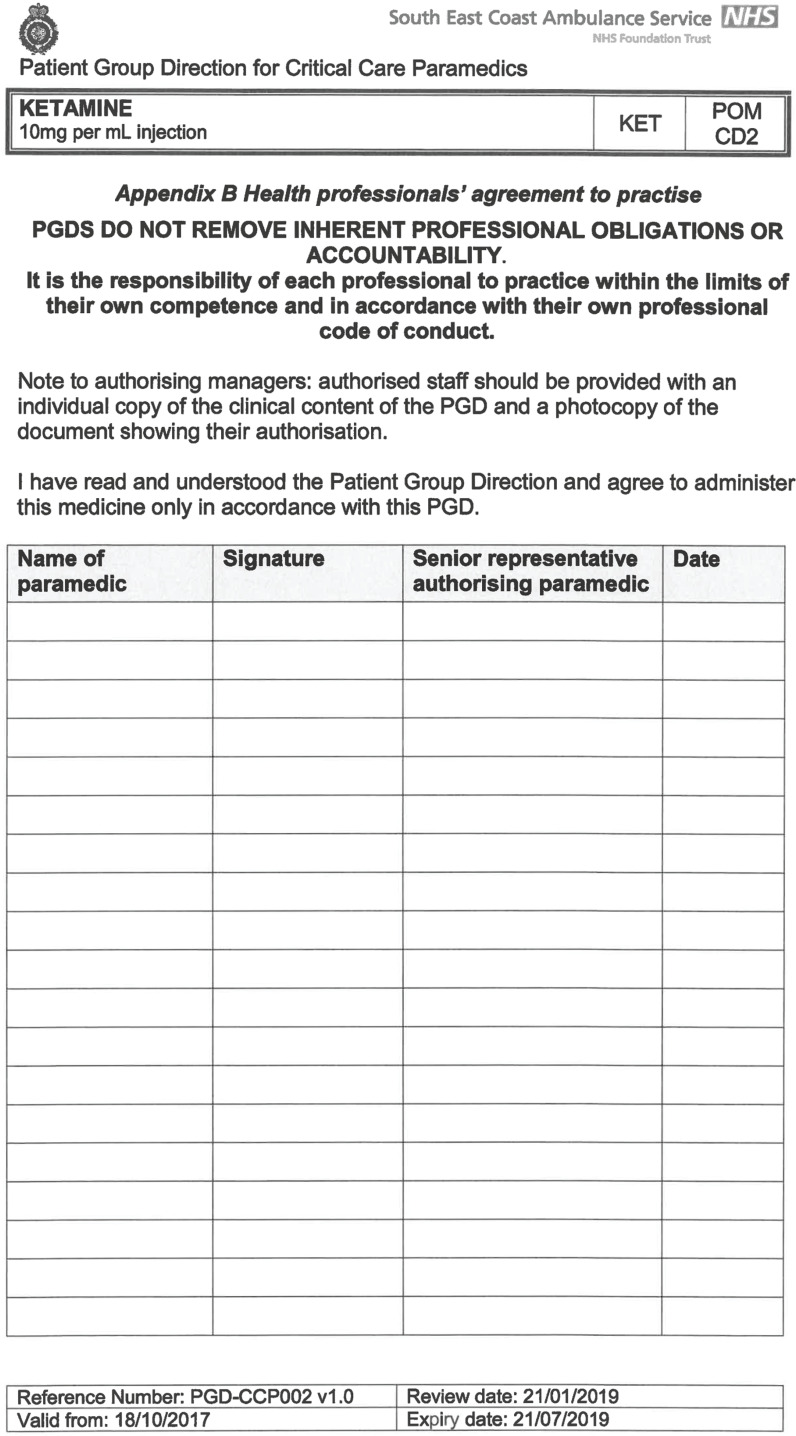



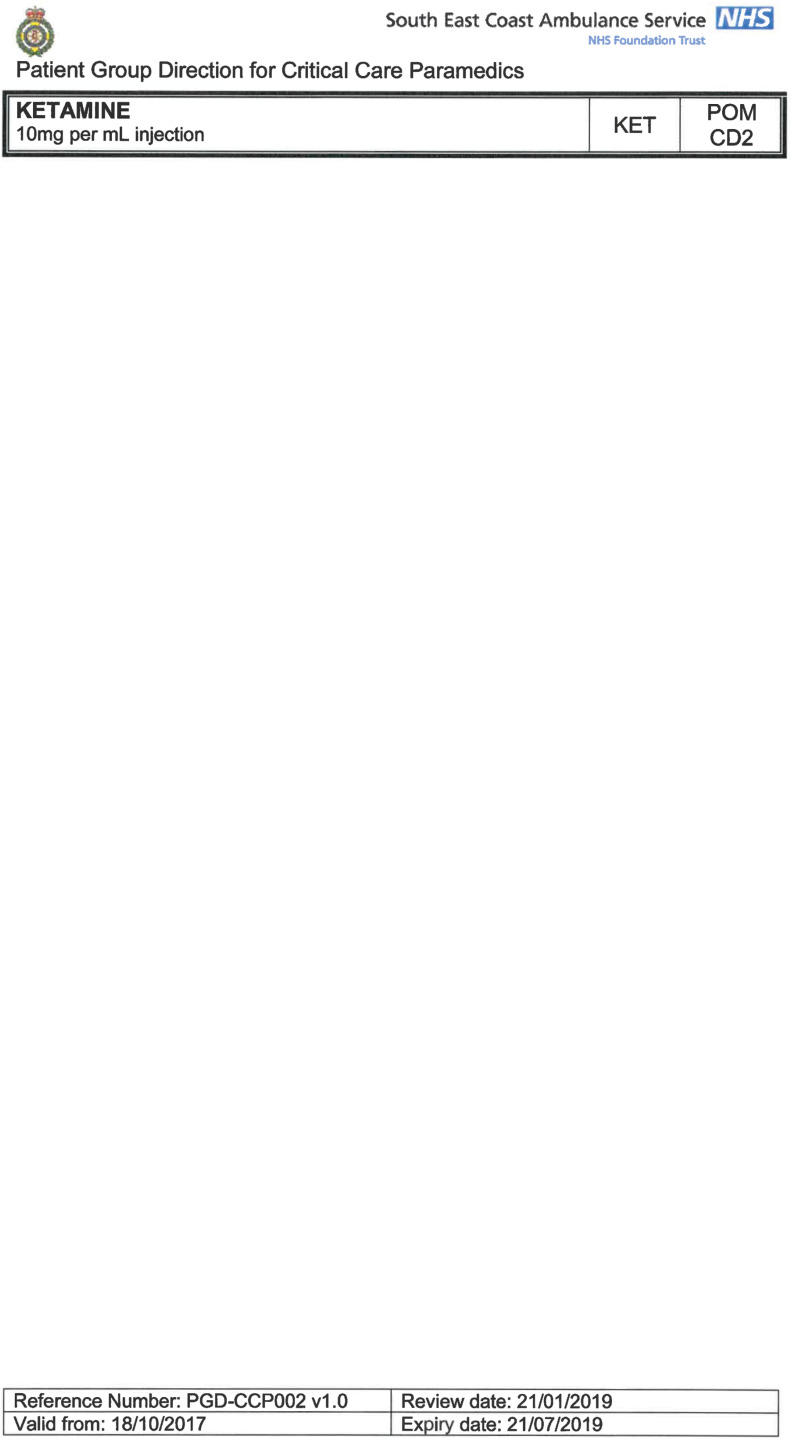

## Introduction

In the national and international pre-hospital setting, ketamine has been used for many years, both in military and civilian medicine. While ketamine has been successfully administered by paramedics in the UK out-of-hospital setting, there is very little information available regarding paramedic use, and it is far less widely reported when compared to physicians (McQueen, Crombie, Cormack, & Wheaton, 2014). Ketamine is not listed in Schedule 17 of the Human Medications Regulations 2012, and as such it is only used by paramedics under a Patient Group Direction (PGD). Some specialist paramedics have locally written PGDs and Clinical Management Plans (CMPs) which allow the administration of ketamine (McQueen et al., 2014; Von Vopelius-Feldt & Benger, 2014). However, nationally, many of these normally operate within, or are aligned to, an air ambulance service.

South East Coast Ambulance Service NHS Foundation Trust (SECAmb) has developed a critical care programme, comprised of experienced paramedics (minimum three years post registration) who undertake a further year of Level 7 postgraduate study, obtaining a Postgraduate Certificate in Patient Assessment and Management (Critical Care). In addition, an enhanced governance system and 24-hour consultant physician support have permitted the administration of ketamine under a PGD (Table 1).

**Table 1.** Basic summary of PGD.

Indications (analgesia)	Management of moderate to severe pain
Indications (sedation)	Life or limb threatening conditions that require sedation for emergency care. Management of the undifferentiated cerebrally agitated trauma patient.
Inclusion criteria (analgesia)	Adults and children aged 12 years and above who are in traumatic pain and other analgesia contra-indicated or have failed to control the pain or the patient has life/limb threatening injuries and the pain is preventing emergency treatment.
Inclusion criteria (sedation)	Adults and children over 12 years of age who require analgesia and sedation to facilitate a life or limb saving intervention such as fracture reduction. Or Adults and children over 12 years of age who are combative and unmanageable due to cerebral agitation after suffering undifferentiated trauma. Another trained pre-hospital care provider competent in airway management must be present at scene.
Exclusion criteria	Known allergy to ketamine or excipients, where essential monitoring (including EtCO2) is not possible, known concurrent theophylline administration, significant hypertension, severe coronary/myocardial disease, severe CVS disease, raised ICP, raised IOP, known history of psychosis, pre-eclampsia, eclampsia, acute porphyria, intracranial mass lesions, hydrocephalus.
Dosing (analgesia)	Initial bolus dose of 0.1 mg/kg; repeat doses of 0.1 mg/kg by titration to achieve intended analgesic effect. For practical purposes: Small adult (40-70 kg): 5 mg aliquots Large adult (≥70 kg): 10 mg aliquots Pain must be reassessed between doses to a maximum dose of 0.5 mg/kg in 30 minutes.
Dosing (sedation)	Initial bolus dose of 0.1–0.2 mg/kg repeated by 0.1–0.2 mg/kg per minute by titration to achieve intended sedative effect. For practical purposes: Small adult (40-70 kg): 5 mg aliquots Large adult (≥70 kg): 10 mg aliquots Pain must be reassessed between doses to a maximum dose of 0.5 mg/kg in 30 minutes.

Note: In order to exceed the maximum dose, a consultant telephone call must be made.

There are currently nine critical care paramedic (CCP) teams distributed across the Trustߣs geographical area. Each geographical team comprises seven CCPs, including two practice leads. Each of the seven CCPs are allocated to one of seven ߢhorizontalߣ teams that meets every seven weeks for a period of shared governance and skills assurance training. This governance time is paramount to ensure patient safety as it allows for a high level of oversight, combined with revalidation training, uplift of new skills and a platform for continued professional development. Each of these sessions is overseen by a CCP practice lead.

Within SECAmb, ketamine is administered as an analgesic, forming part of a multi-modal analgesia approach, and as a procedural sedative in the case of traumatic injury and the management of agitated head injuries. It is also indicated as a sedative where there is a need for transcutaneous pacing or cardioversion, though these latter two indications have not been included in this evaluation (Table 1). Since this evaluation, the SECAmb PGD has been revised and updated (Supplementary 1 and 2). Note that, unlike many international paramedic programmes, ketamine is not currently administered within SECAmb for any kind of acute behavioural disturbance outside the context of traumatic head injury.

Ketamine is only administered by CCPs in SECAmb. In the first year of a CCPߣs practice, ketamine can only be administered following a consultant phone call. If, after this year, competence can be proven both theoretically and in practice, then autonomous administration can commence.

## Methods

### Setting

SECAmb is an urban, suburban and rural NHS funded ambulance service that broadly encompasses the counties of Sussex, Surrey and Kent and receives nearly 862,000 calls each year (South East Coast Ambulance Service NHS Foundation Trust, n.d.). It employs 2700 clinical staff of which 60 (2.2%) were operating as CCPs as of 30 April 2017.

### Patient selection

All patients receiving ketamine for a traumatic aetiology, who had ketamine administered by a CCP, and who had a completed record on the CCP registry of Advanced Life Saving Interventions and Procedures (ALSIP), or on CCPBase (Medic One Systems Ltd), between 16 March 2013 and 30 April 2017, were included. A complete record includes: date of incident, patient age, indication for administration and dose administered. Records were excluded if administration was for a medical aetiology, the ALSIP was incomplete or ketamine was administered by a non-CCP (e.g. a doctor employed by the Trust). All included entries were reviewed for side effects and adverse events that occurred during/after administration, while the patient was still in the care of the CCP.

### Statistical analysis

The data were collated in Microsoft Excel (2010) and analysed using R 3.3.3 (R Core Team, 2017). Following anonymisation of the data (both patient and CCP), descriptive statistics were generated. It was not possible to capture the precise dosing regimen (weight, intervals, etc.) as this was not part of the original dataset.

## Results

A total of 510 unique administrations were identified. Following the exclusion of 61 records (Table 2), 449 (88.0%) administrations remained for inclusion. The most common indication for administration of ketamine was lower limb injury, with 228 (50.8%) administrations (Figure 1 and Table 3). All ketamine was administered intravenously, and the median dose of ketamine for all administrations was 30 mg (interquartile range (IQR) 20–40 mg). The gender split was dominated by males who accounted for 302 (67.3%) of the administrations compared to 147 (32.7%) females (Figure 2). This is in keeping with the most recent trauma epidemiology statistics for the UK (Kehoe, Smith, Edwards, Yates, & Lecky, 2015). The median age of patients was 44 (interquartile range (IQR) 28–58), with women on average being older (median 53, IQR 31–75) than men (median 40, IQR 26–54). Telephone calls to a consultant were made for 243/449 (54.1%) of the administrations, reflecting a need for sanctioning of the drug, advice on dosages or indications, for example.

**Figure:**
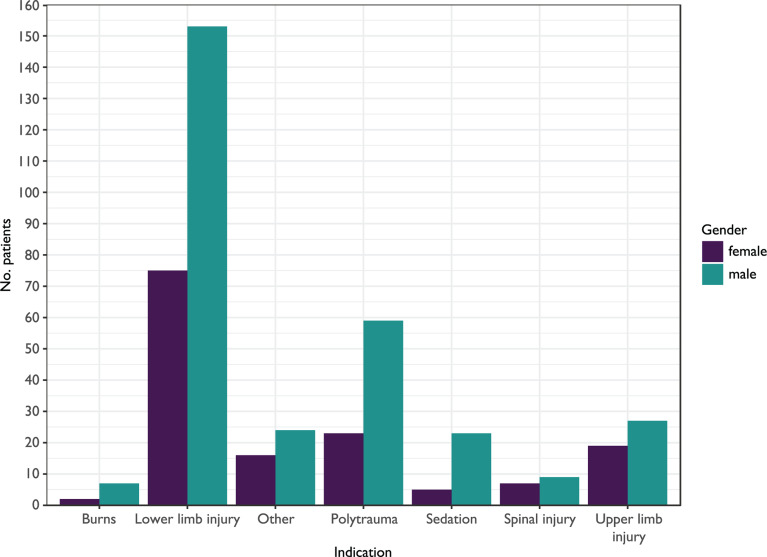
Figure 1. Simple bar chart showing patient counts for each indication, stratified by gender.

**Table 2.** Excluded records with rationale.

Exclusion rationale	Number
Non-traumatic aetiology	27
No complete ALSIP	23
Ketamine drawn up but not administered	8
Ketamine not administered by CCP	1
Duplicate entry	2
**Total**	**61**

**Figure:**
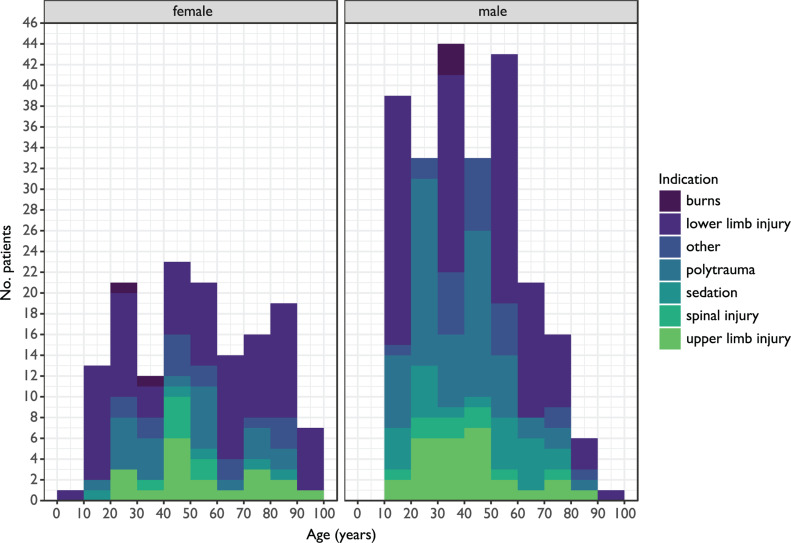
Figure 2. Histogram, comparing age with patient counts. Fill colour shows indication.

**Table 3.** Basic summary table.

Indication	n	Median total dose (mg)	IQR total dose (mg)	Min total dose (mg)	Max total dose (mg)	Male	Female	Median age (years)	IQR age (years)	Min age (years)	Max age (years)
Lower limb injury	228	30	20–40	5	90	153	75	44	25–61.5	7	96
Polytrauma	82	30	20–40	10	80	59	23	40	28–53	18	86
Upper limb injury	46	30	20–40	5	80	27	19	46	33–60	17	91
Other	40	20	10–30	5	180	24	16	44	38.5–57.5	20	88
Sedation	28	22.5	20–35	10	90	23	5	52	25.5–64.5	16	85
Spinal injury	16	22.5	17.5–37.5	10	60	9	7	46	33.5–51	20	78
Burns	9	50	40–70	10	80	7	2	32	30–40	26	47
**Total**	**449**	**30**	**20–40**	**5**	**180**	**302**	**147**	**44**	**28–58**	**7**	**96**

### Side effects and adverse events

Side effects or adverse events were noted in 16/449 (3.6%) of the administrations (Table 4). All of the side effects are in line with the expectancy of known side effects for ketamine analgesia and sedation (Joint Formulary Committee, 2017). No harm was reported in any of the events, and each record documented how the incident was managed by the CCP. This typically involved simple airway manoeuvres, including a period of bag-valve-mask ventilation. Vomiting was not reported in any patient that could not clear their own airway.

**Table 4.** Side effects and adverse incidents as a result of ketamine administration by CCP.

Side effects	n	Median total dose (mg)	IQR (mg)	Minimum total dose (mg)	Maximum total dose (mg)
Transient blood pressure increase	6	30	20–40	15	50
Respiratory depression	3	30	25–35	20	40
Vomiting	3	25	22.5–32.5	20	40
Mild emergence phenomena	2	25	20–30	20	30
Distressed	1	30	30–30	30	30
**Adverse events**					
Accidental OD	1	180	180–180	180	180
**Total**	16	30	20–40	15	180

## Discussion

As far as we are aware, this is the largest published evaluation of out-of-hospital ketamine administration by paramedics. The demographic in this case shows a higher median age and larger proportion of men than most other comparable studies (Bredmose, Lockey, Grier, Watts, & Davies, 2009; Johansson, Kongstad, & Johansson, 2009: Figure 1 and 2; Porter, 2004). Hollis, Keene, Ardlie, Caldicott, and Stapleton (2017) reported a similar age range profile, though again, a higher proportion of women. The reasons for the different demographics here are not known but are likely to reflect the different pre-hospital environments, and the demographics of the local population. The median therapeutic dose is similar to one study (Johansson et al., 2009), though the qualification of administering clinician in that case is unclear. Other studies report a larger mean dose (Bredmose et al., 2009; Porter, 2004), though they are entirely physician delivered. Hollis et al. (2017) describe paramedic administration but do include a mix of IV/IM administration and a number of patients sedated for acute behavioural disturbance. The only other UK based paramedic articles do not mention mean dose (Edwards, Shaw, Gray, Thomson, & Faulkner, 2016; McQueen et al., 2014).

One point of note is that the total doses used in sedation are no different to those used in analgesia. While the PGD recommends a broadly similar dosing regimen for both, intuitively a sedation indication would involve a higher total dose than in analgesia. The most likely explanation for this is that patients who needed sedating are far likelier to have had their care passed to a Helicopter Emergency Medicine Service (HEMS), and so the total dose given by the CCP would appear relatively low in comparison to those patients who remained with the CCP for their entire pre-hospital phase.

Regarding the site of injury, the results also show that isolated limb injuries were by far the most common indication for ketamine analgesia, and considering Lee and Porterߣs (2005) discussion around how common this type of injury is in pre-hospital care, and the need for early fracture reduction, this is unsurprising.

### Side effects

Side effects are reported in most other studies (Bredmose et al., 2009; Edwards et al., 2016; Galinski et al., 2007; Johansson et al., 2009; Porter, 2004; Svenson & Abernathy, 2007) but sample numbers are too low to draw conclusions. Svenson and Abernathy (2007), Porter (2004) and Edwards et al. (2016) report no adverse effects, while Bredmose et al. (2009) reported a similar proportion to this project. None of these categorised adverse events in the same way, but the proportions observed here are within the range reported in the BNF (Joint Formulary Committee, 2017). No other study reported a medication error but, again, no other study has such a large population size. It should be noted that, even at the size of this population, the frequency of reported side effects was low.

When examining the side effects seen in this evaluation within the context of those stated in the BNF (Joint Formulary Committee, 2017), all except respiratory depression fall in to the *common or very common* category (defined as occurring in 1:100 (1%) to 1:10 (10%) of patients), with respiratory depression occurring *rarely* (1:1000 (0.1%) to 1:100 (1%)). If we take the halfway points of these groups (i.e. 5% and 0.5%), then the number needed to harm can be calculated as 35.7 patients for the *common to very common* side effects and 1000 patients for the rare side effects.

The side effects that occurred in this evaluation, while causing no harm to the patient, have the potential to cause complications if not managed correctly. It seems prudent that ketamine continues to be administered only by specialist paramedics, who have received additional, specific training in identifying and managing side effects from the drug, in keeping with the Academy of Medical Royal Colleges (2013) recommendations.

Ketamine use within SECAmb has increased year-on-year, though this is likely to be due both to an increasing scope of practice and numbers of CCPs. The increase may also be due, in part, to improved familiarity with the drug and confidence to administer.

Looking to the future, there may be a role for ketamine use in acute behavioural disturbance, an indication that is commonplace in other parts of the world, and was an indication in the Hollis et al. (2017) study.

### Strengths and limitations

This was a retrospective analysis, strengthened most by its large patient population in comparison to other studies of paramedic administered ketamine. However, care must be taken in generalising these results to other clinical areas, trusts or patient populations, but it should serve as a base for further prospective studies and service development.

Side effects and adverse events did not have a formal method of being recorded, other than in the free text of the report or patient record. Consequently, there is a possibility that side effects or adverse events occurred but were not documented. CCPs are mandated to document any side effects or adverse events, so it is probable that the number of undeclared side effects is low. All CCPs were made aware of the intended study, prior to data collection, so they could retrospectively enter any adverse effects that had not been previously noted, though the authors accept the limitations here that people may not sufficiently recall incidents that are in the past or could have edited them to appear more favourable. Other interventions and therapies were not gathered as part of this analysis. Patients are likely to have received other analgesics, such as morphine, which may bias the dose and side effect profile. The use of anti-emetics is also not recorded within this analysis.

Since methods and detail of data collection changed over the years, interrogation of all entries took place but it was not always clear whether the administration was for analgesia or sedation. Clearly this is another limitation of the study, and future analysis should be clear to separate the two as the dosing regimen is likely to be different.

## Conclusion

Ketamine has been administered over 500 times by CCPs operating within the South East Coast Ambulance Service NHS Trust area. In the largest retrospective analysis of its kind, and one of very few focusing on paramedic only administration, levels of side effects and adverse events are in line with nationally documented expectations. Patient demographics show an increased age when compared with other studies, and a larger proportion of males. These statistics are in line with epidemiological studies in traumatology, and are likely to reflect the traumatic nature of the indications for use, and the older demographic of the geographical region in which the CCPs operate.

This evaluation provides a useful platform for increasing the evidence base for paramedic administered ketamine. However, prospective studies are now needed in order to confirm the safety and efficacy of ketamine administration among the advanced paramedic population.

## Acknowledgements

The authors would like to acknowledge Professor Richard Lyon for his advice and assistance with several elements of the project, and Richard Pilbery for his assistance in generating the statistics and final edit.

## Author contributions

All named authors met the *British Paramedic Journal* guidelines for authorship. In addition:

AC/NG/AW were principally involved with data acquisition, initial design and drafting.

AC/PW/NG/JW/FM were involved with data interpretation, drafting and critical revision.

## Conflict of interest

While there are no formal competing interests to declare, AC/PW/FM are all employed by the Trust at the time of publication.

## Ethics

This study met UK Health Research Agency criteria for a service evaluation. All the data utilised for this study were routinely collected as part of standard pre-hospital patient data collection, sanctioned by the CCP lead, chief pharmacist and Trust medical director. Formal ethical approval was therefore not required.

## Funding

None.
